# Evaluation of the EsteR Toolkit for COVID-19 Decision Support: Sensitivity Analysis and Usability Study

**DOI:** 10.2196/44549

**Published:** 2023-06-27

**Authors:** Rieke Alpers, Lisa Kühne, Hong-Phuc Truong, Hajo Zeeb, Max Westphal, Sonja Jäckle

**Affiliations:** 1 Fraunhofer Institute for Digital Medicine MEVIS Bremen Germany; 2 Department of Prevention and Evaluation Leibniz Institute for Prevention Research and Epidemiology - BIPS Bremen Germany; 3 Fraunhofer Institute for Industrial Mathematics ITWM Kaiserslautern Germany; 4 Fraunhofer Institute for Digital Medicine MEVIS Lübeck Germany

**Keywords:** COVID-19, public health, decision support tool, sensitivity analysis, web application, usability study

## Abstract

**Background:**

During the COVID-19 pandemic, local health authorities were responsible for managing and reporting current cases in Germany. Since March 2020, employees had to contain the spread of COVID-19 by monitoring and contacting infected persons as well as tracing their contacts. In the EsteR project, we implemented existing and newly developed statistical models as decision support tools to assist in the work of the local health authorities.

**Objective:**

The main goal of this study was to validate the EsteR toolkit in two complementary ways: first, investigating the stability of the answers provided by our statistical tools regarding model parameters in the back end and, second, evaluating the usability and applicability of our web application in the front end by test users.

**Methods:**

For model stability assessment, a sensitivity analysis was carried out for all 5 developed statistical models. The default parameters of our models as well as the test ranges of the model parameters were based on a previous literature review on COVID-19 properties. The obtained answers resulting from different parameters were compared using dissimilarity metrics and visualized using contour plots. In addition, the parameter ranges of general model stability were identified. For the usability evaluation of the web application, cognitive walk-throughs and focus group interviews were conducted with 6 containment scouts located at 2 different local health authorities. They were first asked to complete small tasks with the tools and then express their general impressions of the web application.

**Results:**

The simulation results showed that some statistical models were more sensitive to changes in their parameters than others. For each of the single-person use cases, we determined an area where the respective model could be rated as stable. In contrast, the results of the group use cases highly depended on the user inputs, and thus, no area of parameters with general model stability could be identified. We have also provided a detailed simulation report of the sensitivity analysis. In the user evaluation, the cognitive walk-throughs and focus group interviews revealed that the user interface needed to be simplified and more information was necessary as guidance. In general, the testers rated the web application as helpful, especially for new employees.

**Conclusions:**

This evaluation study allowed us to refine the EsteR toolkit. Using the sensitivity analysis, we identified suitable model parameters and analyzed how stable the statistical models were in terms of changes in their parameters. Furthermore, the front end of the web application was improved with the results of the conducted cognitive walk-throughs and focus group interviews regarding its user-friendliness.

## Introduction

### Background

In Germany, the local health authorities played a central role in the management and containment of the COVID-19 pandemic. The employees ordered isolation for infected persons and traced contact persons to quarantine them according to the COVID-19 regulations of the Robert Koch Institute (RKI). The occurring infections were reported by each local health authority to the RKI. During the COVID-19 pandemic, the local health authorities focused only on the management of the pandemic, and during periods of high infection numbers, they were even incapable of conducting contact tracing [1].

The efficiency of processes in local health authorities can be increased using decision support tools. In other working areas, such decision support models have already been developed. For example, in hospitals, decision support tools are used to streamline the diagnosis and prognosis of COVID-19 cases [2]. Furthermore, governments can use models for predicting the number of COVID-19 infections to adapt their current COVID-19 regulations [3].

In the EsteR project, we developed a toolkit for local health authorities to assist with their work. It includes statistical models for 5 specific use cases and a web application to support isolation and quarantine decisions. The 3 use cases for infected persons include the infection period, the time when they were most likely infected; the illness period, the time range when their infected contacts will most likely start to show symptoms; and the infectious period, the time when they are most likely infectious to others. Furthermore, statistical models for 2 group use cases were introduced: a model for estimating the further expected infections from a group event after some attendees develop COVID-19 symptoms and a model for calculating the probability that nobody was infected in a childcare group or school class with one or more index cases and only negative results for tested susceptible group members. For each use case, a statistical model was developed based on the COVID-19 properties reported in the literature. The entire toolkit was already introduced in detail in the article by Jäckle et al [4].

### Objectives

In this paper, we presented two complementary evaluations of the EsteR toolkit. First, a sensitivity analysis of the model parameters was carried out. In research, sensitivity analyses have been recently used to analyze the COVID-19 transmission dynamics and which regulations allow for control of the pandemic [5-9]. For our statistical models, the aim of the sensitivity analysis was to determine how a variation in the model parameters influences the answers provided in the tools. In the second step, the usability and applicability of the web application were evaluated. Cognitive walk-throughs analyzed the usability of applications for certain tasks and revealed improvements for easy and efficient use as it has already been shown for digital applications targeting health outcomes (eg, disease self-management tools [10,11]). The aim of this study’s cognitive walk-throughs was to identify critical usability problems and generate recommendations to improve the efficiency and user acceptance of the tool. In addition, focus group interviews were conducted, which are often applied for implementing and evaluating public health interventions (eg, to elaborate on users’ expectations of clinical decision support tools [12,13]). We aimed to reveal containment scouts’ perceptions and whether the web application was supportive of their decision tasks.

To our knowledge, this is the first evaluation of a support tool for local health authorities for assessing personal transmission risks. It can support other researchers by serving as an example for evaluations of two important aspects: the statistical models in the back end and the usability in the front end. Our sensitivity analysis demonstrates how the implications of a choice of study can be assessed and how the results can be interpreted from the perspective of real-world implications. We support reproducibility by providing all data and code for the analysis in a public GitHub repository. The usability evaluation showed how the combination of cognitive walk-throughs and focus group interviews helped depict the varying requirements of users with different professional backgrounds. Unlike a clinical decision support tool used only by medical personnel, the staff of local health authorities during the COVID-19 pandemic mostly had only minor previous knowledge in virology and had to deal with a hitherto unknown situation and accompanying tasks.

## Methods

### Sensitivity Analysis for Statistical Models

#### Overview

We conducted a simulation by varying the model parameters of the 5 statistical models while keeping the input data fixed. The outcomes obtained using each new parameter set were compared with those obtained using the default values. For the incubation time (used for the infection period and infection spread use case) and the serial interval (used for the illness period use case), the literature review described in the study by Kühne et al [14] identified more than one study reporting suitable parameters. Our selection approach to the default parameters for our web application is described in the first chapter of Multimedia Appendix 1 [15-74] and visualized in Figures S1 and S2 in Multimedia Appendix 1. Table 1 summarizes the main aspects of the simulation. In the following section, we briefly explain each use case, where each subsection corresponds to one column in Table 1. Afterward, we describe the dissimilarity metrics used to compare the outcomes and how we visualized the results. For more details, see also the second chapter of Multimedia Appendix 1. The code and extracted study data can be found on GitHub [75].

**Table 1:** Overview of the simulation strategy for the sensitivity analysis of the 5 statistical models.^a^

	Infection period	Infection spread	Illness period	Infectious period	Group quarantine
COVID-19 property	Incubation time	Incubation time	Serial interval	Infectious period	Setting-specific transmission
Distribution	Lognormal	Lognormal	Gamma	Gamma	Beta binomial
Studies	[15,19-45]	[15,19-45]	[16,19,30,34,36,44-73]	[17]	[18]
Model parameters	E(X)^b^ μ*^c^	E(X) μ*	E(X) SD(X)	E(X) SD(X)	P(K>0)^d^ E(K|K>0)^e^
Default values (parameter ranges)	6.3 (3.9-9.1) 5.4 (3-8.5)	6.3 (3.9-9.1) 5.4 (3-8.5)	5.54 (2.51-8.72) 3.9 (1.21-5.65)	12.89 (8-18) 2.84 (1-5)	0.3 (0.24-0.37) 3.3 (1-8)
Outcome	80% HDR^f^ distribution	Expected infections	80% HDR distribution	80% HDR distribution	Probability of no infection
Metric	1-IoU^g^ W1^h^	Δpred^i^	1-IoU W1	1-IoU W1	Δprob^j^

^a^For each use case, the COVID-19 property and the distribution type for modeling it, the number of studies reporting suitable parameters, the respective 2 model parameters with their respective default value and the assessed range, and the considered model outcomes and their respective metrics are listed.

^b^E(X): mean.

^c^μ*: median.

^d^P(K>0): transmission probability.

^e^E(K|K>0): conditional mean of infected persons in case of transmission.

^f^HDR: high-density region.

^g^IoU: intersection-over-union.

^h^W_1_: Wasserstein metric.

^i^Δpred: difference in predicted infections.

^j^Δprob: difference in probability.

#### Simulation Strategy

##### Infection Period

The underlying distribution in the infection period model was a lognormal distribution for the incubation time [4,15]. For the simulation, the mean parameter μ and the SD parameter σ for a reasonable lognormal distribution were calculated using the lognormal distribution properties for the median μ* and mean *E*(X), namely, μ* = *e*^μ^ and 
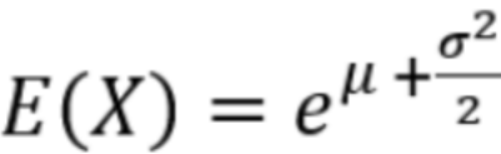
. In the simulations, a scenario of one infected person starting to show symptoms on March 22, 2022, was used. We defined a grid of 100 equidistant values, each from the smallest to the largest reported mean and median identified in the study by Kühne et al [14], and calculated the distribution and the 80% high-density region (HDR) of the infection period of the infected person for each parameter set and the default values.

##### Infection Spread

This model was built using the same incubation time distribution as that of the infection period model. Hence, we used the same parameter ranges in the simulation. A group event scenario with 20 attendees on March 22, 2022, with 3 persons starting to show symptoms until March 26, 2022, was considered. Using the same equidistant parameter sets and default values as for the infection period use case, the total number of expected infections from the group event was calculated.

##### Illness Period

The illness period of contact persons was modeled using a gamma distribution of the serial interval [4,16]. For this use case, we used the articles from the literature review that reported the mean *E*(*X*) and *SD*(*X*) of the serial interval. We calculated α and β using the gamma distribution properties 
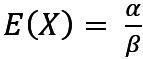
 and 
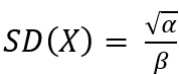
. By choosing the gamma distribution, we implicitly assumed that the serial interval was strictly positive. For the simulation, a scenario with one infected person starting to show symptoms on March 22, 2022, was considered. As with the infection period, a grid of 100 equidistant values for each parameter was defined. All parameter combinations and the default values were used to calculate the distribution and the 80% HDR of the symptom onset period for the first, second, and third contact generation.

##### Infectious Period

For modeling the infectious period, only one study was available that directly used a gamma distribution [4,17]. For comparability across the use cases, we calculated the mean and SD of that gamma distribution, as described previously, and defined their own ranges to test them using a similar size as in the illness period use case. As before, a scenario with one infected person starting to show symptoms on March 22, 2022, was considered, and a grid of 100 equidistant values for each parameter was defined. The default parameters and all combinations from that grid were used to calculate the distribution and the 80% HDR for the infectious period.

##### Risk Assessment for Group Quarantine

In the group quarantine use case, the 2 parameters α and β needed for the beta binomial prior distribution were determined based on the probability *P*(*K*>0) that at least one person was infected and the conditional mean number of infected persons in the case of transmission *E*(*K*|*K*>0) [76]. The web application provides a calculation of the probability of no further infections in 2 settings: a childcare group and a school class. Table 1 lists the values for the childcare setting; for the school class, we used default values of *P*(*K*>0)=0.12 and *E*(*K*|*K*>0)=1.77 in the EsteR toolkit and conducted the sensitivity analysis for *P*(*K*>0) in the range from 0.05-0.21 and for *E*(*K*|*K*>0) in the range from 1-8. In both settings, the ranges of the transmission probability were based on the reported 95% CIs [4,18].

In the simulation, we used 2 typical scenarios for our settings: a childcare group of 14 persons plus 1 index case and a school class of 27 persons plus 2 index cases. For both scenarios, a grid of 100 equidistant values for each parameter and the default values were used to calculate the probability of no further infection based on the changed prior. Furthermore, we conducted a sensitivity analysis for the diagnostic test sensitivity, which influenced the likelihood of the probability calculation. A consistent shift in the range of –0.2 to 0.2 of the day-specific polymerase chain reaction test sensitivity provided by Kucirka et al [74] over all days was simulated, and the probability of no further infections in the childcare scenario was calculated.

#### Dissimilarity Metrics

For the infection period, illness period, and infectious period use cases, the web application provides an answer sentence containing the 80% HDRs as a range of dates accompanied by a plot of the distributions highlighting the 80% HDRs. Thus, the goal was to test how these outputs change with different model parameters. We decided to compare the 80% HDRs of 1 parameter set (HDR_1_) with the 80% HDRs resulting from the default values (HDR_2_) using an inverted version of the intersection-over-union (IoU) metric [77]:



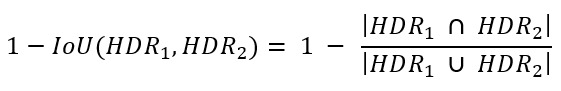



By inverting the IoU metric, a metric value of 0 indicates a perfect equality of 2 outcomes, and a metric value of 1 corresponds to the situation in which the 80% HDRs do not overlap at all.

These distributions were compared using the Wasserstein metric with *p*=1 [78], which is defined as 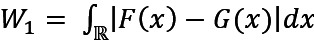 where *F* and *G* denote the cumulative distribution functions of their respective distributions. As the discretized versions of the distributions were used in our calculations, we also estimated the integral as a standardized sum of differences. With *W*_1_, the changes in the distribution tails were also considered. Although the minimum of 0 of the Wasserstein metric indicates equal distributions, there is no upper bound for the metric as the masses of the 2 distributions can be arbitrarily far away from each other.

For the infection spread, the difference Δ*pred* between the number of total symptomatic infections predicted using simulation parameters and predicted using default parameters was determined. In the last use case, the change in the probability of no further infections was calculated as the difference Δ*prob* between the probability calculated using simulated and default parameters. Both differences allow for easy and direct interpretation of the changes in the respective outcomes.

#### Visualization

For all simulations—except the test sensitivity evaluation of the last statistical model—a contour plot was created showing the resulting values; the currently used parameters as red squares; and contour lines with step size 0.2 for the 1-IoU plots, 1 for the *W*_1_ plots, and 0.05 for the Δ*prob* plot. In the infection period plot, no contour lines were shown as the Δ*pred* values were integers. Furthermore, where available, the published parameter combinations were visualized as different marker shapes according to their period of data collection. For each statistical model, we included the most relevant plot in this paper. All the plots as well as a more detailed description can be found in the third chapter of Multimedia Appendix 1.

### Usability Evaluation

#### Cognitive Walk-Through

For the usability evaluation, we conducted cognitive walk-throughs based on the theory by Lewis and Wharton [79]. The process of a cognitive walk-through includes the definition of a sample task that potential users try to solve. This action sequence is then analyzed to provide feedback to the developers about the users’ ability to perform tasks using the web application and which actions likely result in operating errors. The focus was on understanding the learnability of the system for new or occasional users.

For the evaluation, we performed cognitive walk-throughs with potential end users of the web application. The tests included example tasks on contact tracing, one test for each of the 5 use cases (Textbox 1).

The testers were encouraged to speak their thoughts out loud while using the web application. In addition to the example tasks, we asked questions about the usability and applicability of the EsteR toolkit (eg, about the orientation on the starting page and within each tool), as well as the amount and level of detail of the inputs and results. The comprehensiveness of the visualizations was of special interest as these results are the basis for deciding on further handling of the case or contact person. The users were asked how difficult it was to complete the tasks. A 5-point Likert scale was presented indicating the users’ impressions of the web application. The describing attributes included disturbing or supportive, complicated or simple, inefficient or efficient, confusing or clear, and uninteresting or interesting, with the possibility to rate on the scale between these opposites. Moreover, the users were encouraged to provide general feedback. The test was audio recorded, transcribed, and analyzed to provide improvements for the user interface (UI) and suggest a guideline for use.

**Example tasks on contact tracing for each use case.**

1. A person reports the first symptoms on 17.05. On 14.05, the person was at an event. How do you rate the chance that the person got infected there?
2. A mother reports the first symptoms of her children on 18.05. When can she expect to become ill herself?
3. A person reports the first symptoms on 13.05. On 10.05, she went on a trip with 3 friends and they sat together in the car for half an hour. How do you rate the chance that the friends got infected?
4. On 14.05, a group of 15 adults met. The meeting was held in a closed room and no masks were worn. One person reported the first symptoms on 17.05. How do you rate the chance that people got infected at the meeting?
5. Over the weekend, 2 students of a class became ill. The school class of 22 persons met last on 13.05. The infected students were able to contact a few classmates, so 4 tested negative on Sunday. On Monday, the remaining children were given an antigen test, which was negative for all of them. How do you rate the chance that no other children in the class will get sick?

#### Focus Group Interviews

Focus group interviews are often conducted in public health research as they allow for an exploration of the attitudes, perceptions, and ideas of the target group regarding a topic. The focus group consists of a small group of people with common characteristics, in our case, containment scouts at a local health authority in Germany. The interview is designed to allow for a reflection on the questions asked by the interviewer and a discussion of different answers in the group [80].

We conducted 2 independent focus group interviews with representative containment scouts of the cooperating local health authorities in Berlin-Reinickendorf and Bremerhaven. The containment scouts were asked to share their perceptions in more detail after they conducted the cognitive walk-through and, thus, gained firsthand experience with the web application. The focus was on identifying potential implementation barriers in the local health authorities as well as technical and ergonomic requirements. The containment scouts were asked to rate the effort and benefits of using the web application in their daily work and for additional tasks possibly coming up in the course of the pandemic. The interviews were audio recorded and transcribed. The results were obtained through qualitative content analysis in addition to the impression of the interviewers.

### Ethical Considerations

The study participants were recruited from the local health authorities that were already cooperating with the EsteR project during the toolkit development. Before participant recruitment, the local health authorities were given written information about the status of the toolkit and the objectives of its evaluation. Moreover, the study participants were verbally informed about the toolkit and the procedures of the cognitive walk-through and the focus group. All participants gave their consent to take part in the cognitive walk-through and the interviews and agreed to the audio recording. The data were collected, stored, and processed anonymously. The study did not seek ethics approval as the collected data referred to workflows in the specified work setting only and were not considered sensitive to participants’ personal interests.

## Results

### Sensitivity Analysis for Statistical Models

#### Infection Period

**Figure 1:**
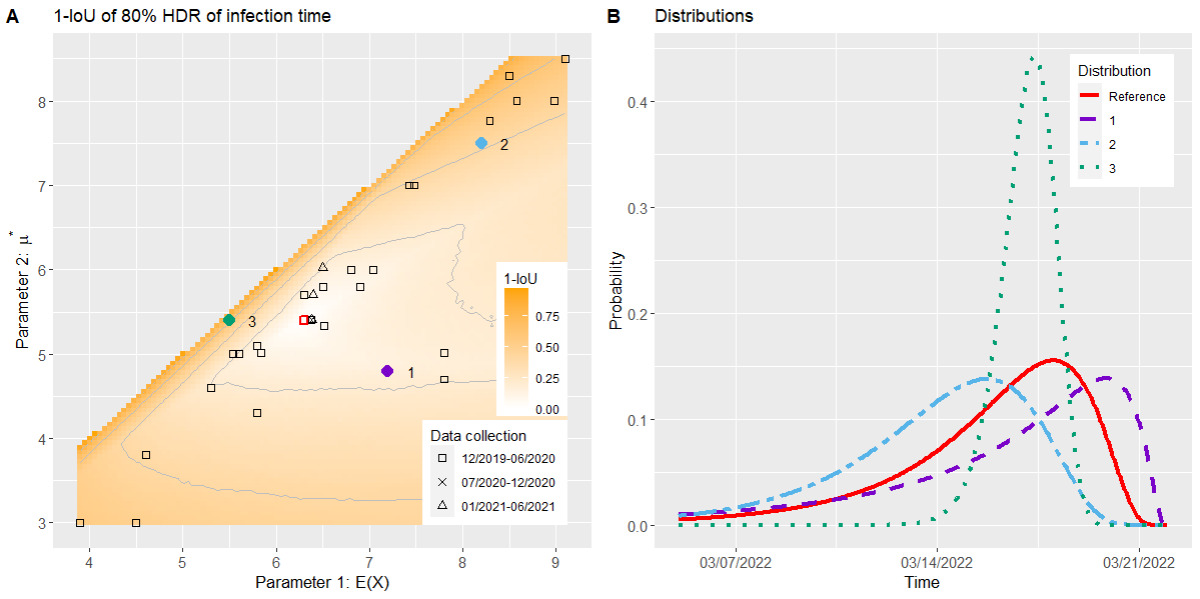
Simulation result of the infection period model: 1–intersection-over-union (IoU) of the 80% high-density region (HDR) of the infection time (A) and the distributions derived from 4 specific parameter sets (B). The default parameters are marked in red.

The included studies for the incubation time gathered around a line with μ* ≈ *E*(*X*) – 1, recognizable in Figure 1A (corresponding to Figure S3 in Multimedia Appendix 1), and only 7% (2/28) of studies below this line could be considered outliers. All 3 (11%) studies with data from the second half of 2020 and the first half of 2021 were concentrated close to our default model parameters.

For the infection period, the 1-IoU of the predicted 80% HDRs showed small values along 3 directions starting from the default values: first, toward the lower left corner along the included studies; second, almost horizontally through the 2 outlier studies to the right; and, finally, upward to the right but staying below the upper included studies. The highest 1-IoU values were found along the diagonal, where μ* ≈ *E*(*X*). In the other areas of the plot, the values remained mainly <0.6, and approximately half (15/28, 54%) of the studies lay inside the first contour line of 0.2.

In general, the Wasserstein metric exhibited a similar behavior regarding areas of high and low values (Figure S4 in Multimedia Appendix 1).

The distributions of the 3 parameter sets with different 1-IoU values, together with the distribution from our default parameters, are plotted in Figure 1B. For the lognormal distribution, the density had a narrow, high peak when the mean and median were close together, as can be observed for point 3. In contrast, when the mean and median were farther apart, the SD increased and the peak became smaller. When the mean and median were shifted by the same amount, the distribution width remained intact but was rather shifted. For the parameter set of point 1, the skew to the right of the lognormal distribution increased, whereas for point 2, it decreased. Hence, the Wasserstein metric of point 1 was relatively high compared with that of the 1-IoU, whereas both metrics behaved similarly for point 2.

#### Infection Spread

**Figure 2:**
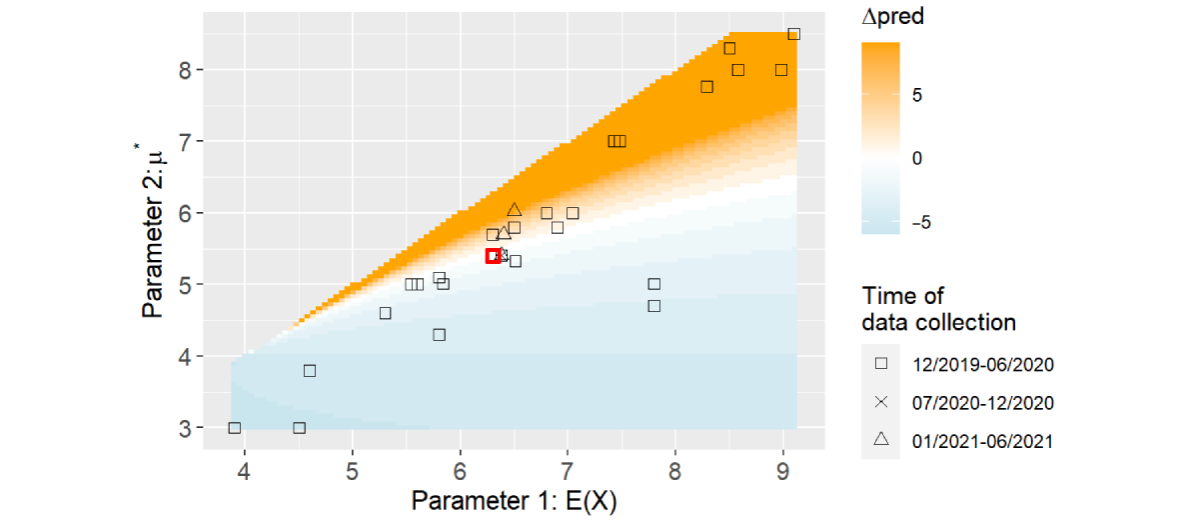
Simulation result of the infection spread model: difference in the predicted total infections after a group event. The default parameters are marked in red.

For the infection spread model, the higher the median or the lower the mean incubation time, the higher the predicted total infections were. However, the change in prediction was far more distinct for the median, which is visible in Figure 2 (Figure S5 in Multimedia Appendix 1). No change in prediction could be observed along the line through the default parameters, with a slope of approximately 0.4. The maximum value of 9 could be found close to the diagonal with μ* ≈ *E*(*X*) fanning out toward the top, whereas the minimum of –6 was located in the lower left corner. All prediction differences were ≤9 because, in the considered setting, the prediction from the default values was 11, and all predictions were limited upward by the total group size of 20. As only mean and median incubation times of at least 3.9 and 3, respectively, were tested and a scenario with 3 observed illnesses after 4 days was considered, the minimal predicted number of total infections was 5, and the minimal difference from the default prediction was –6. The predictions were rounded upward, which resulted in the discrete differences and stepwise changing colors.

#### Illness Period

**Figure 3:**
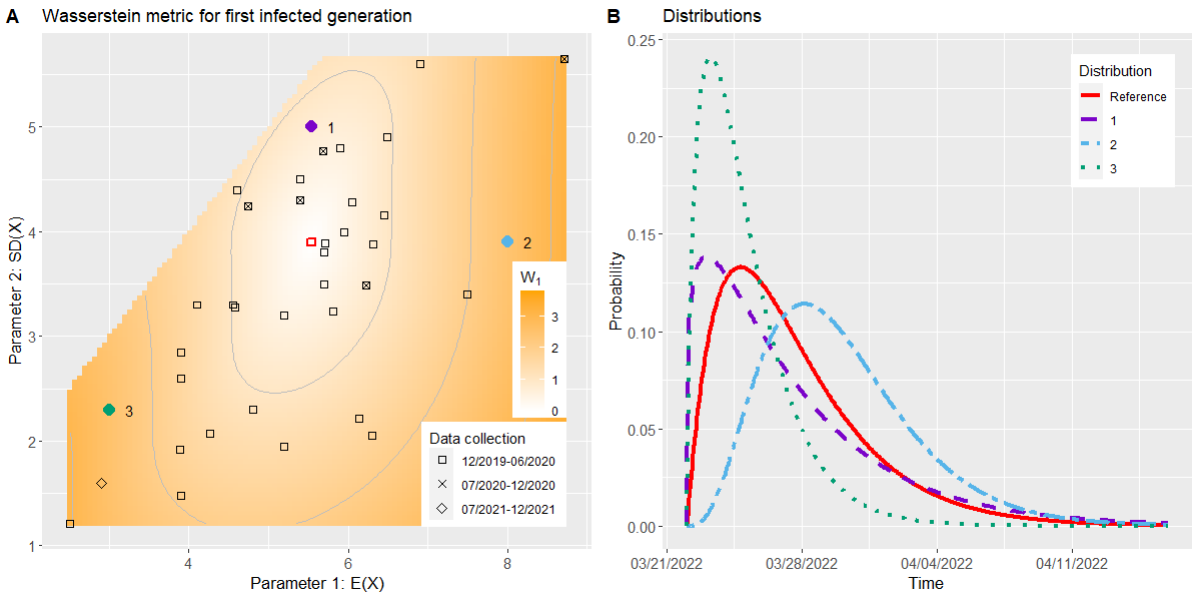
Simulation result of the illness period model: Wasserstein metric for the distribution of the symptom onset of the first contact generation (A) and the distributions of 3 specific parameter sets (B). The default parameters are marked in red.

As shown in Figure 3A, the included studies for the serial interval spread more than the studies for the incubation time, but they mainly remained within the range of *SD*(*X*) ∈ [*E*(*X*) – 3, *E*(*X*) – 0.5]. Only a small number of studies (5/35, 14%) were outside this range and, thus, far away from the default parameters.

In all 3 generations, the 1-IoU of the 80% HDRs of the symptom onset followed a pyramidal course (Figures S6-S8 in Multimedia Appendix 1).

For the Wasserstein metric, Figure 3A (corresponding to Figure S9 in Multimedia Appendix 1) shows that the course of the values has an oval shape stretched vertically. Along the mean, the increase in the Wasserstein metric was not as high as that of the SD. The highest values could be found on the left and right sides of the plot, with a maximum of approximately ≥3. A total of 54% (19/35) of the included studies were within the contour line with a value of 1. For the second and third generations, the shape of the plot remained the same, whereas the horizontal incline increased up to double its original value, as can be seen in Figures S10 and S11 in Multimedia Appendix 1.

The distributions of 1 parameter set with a low Wasserstein metric and 2 with high Wasserstein metrics for the first generation, together with the distribution resulting from our default parameters, are plotted in Figure 3B. Increasing the SD while maintaining the mean shifted the mode and made the distribution more skewed, whereas most of the mass remained in place. When the mean increased, the main mass of the distribution shifted to the right and the skew slightly decreased. For a low mean and SD, the distribution was very narrow and had a high peak around the mean. Hence, both the 1-IoU and the Wasserstein metric were relatively low for point 1 and high for points 2 and 3. In a joint alteration of *E*(*X*) and *SD*(*X*), the effects may have partly canceled each other out, and the main changes happened in the tails of the distribution. This explains why the 1-IoU followed a pyramidal course, whereas the Wasserstein metric had a vertically stretched oval shape. This effect was strengthened over different generations as, with the convolution we used in our model, the first parameter of the gamma distribution was multiplied by the generation number.

#### Infectious Period

**Figure 4:**
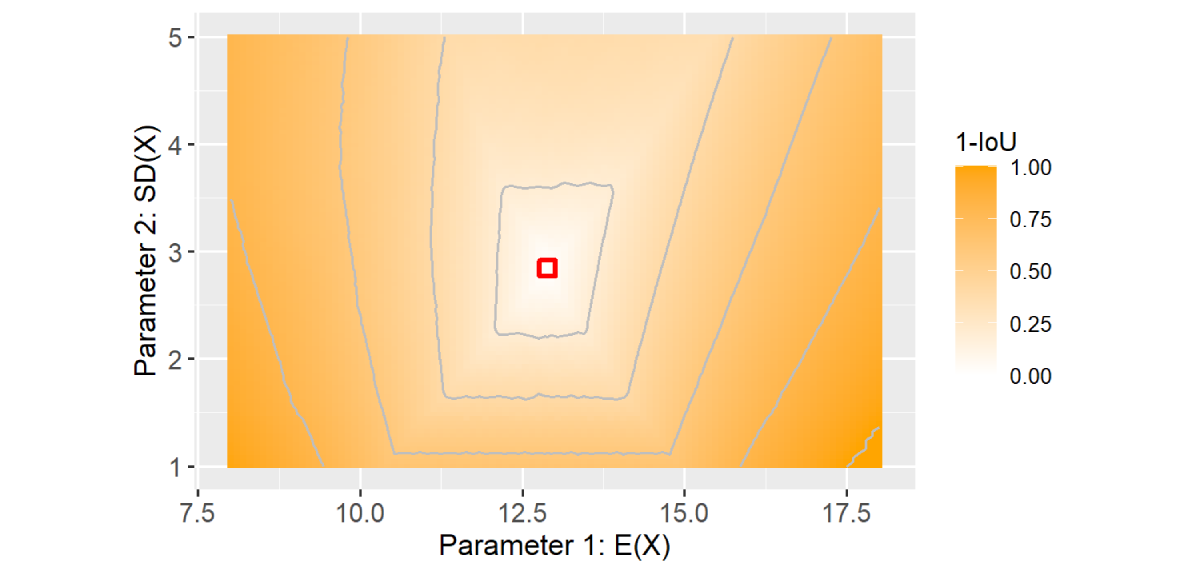
Simulation result of the infectious period model: 1–intersection-over-union (IoU) of the 80% high-density region of the infectious period. The default parameters derived from data from the first half of 2020 are marked in red.

Both the 1-IoU of the 80% HDRs and the Wasserstein metric plots showed a similar behavior as that of the illness period model as it also used a gamma distribution. The pyramidal shape of the 1-IoU is shown in Figure 4 (Figure S12 in Multimedia Appendix 1), where the highest values of ≥0.8 are in the lower left and right corners. With changes of up to 0.6 in both directions in the mean and SD, the 1-IoU remained <0.2. For the Wasserstein metric plot, see Figure S13 in Multimedia Appendix 1.

#### Risk Assessment for Group Quarantine

**Figure 5:**
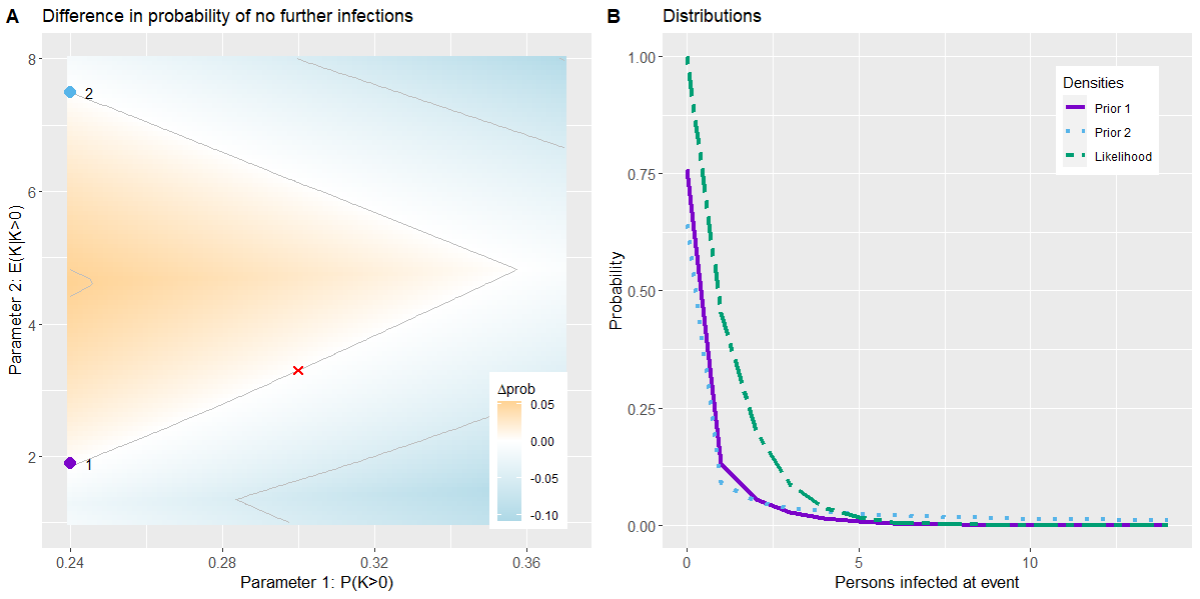
Simulation result of the risk assessment for group quarantine model for the childcare scenario (A) and the prior distributions for 2 certain parameter sets and the likelihood (B). The default parameters derived from data from the second half of 2020 are marked in red.

Figure 5A (corresponding to Figure S14 in Multimedia Appendix 1) shows the results of the childcare scenario. In the part of the plot with *E*(*K*|*K*>0) ∈ [1.5, 4.7], the predicted probability of no further infections increased with a decreasing transmission probability *P*(*K*>0) and an increasing conditional mean *E*(*K*|*K*>0). A white line can be detected, which represents the parameter sets with the same probability as the default set. This part was mirrored at the horizontal lines with conditional mean≈1.5 and 4.7, below and above which the predicted probability decreased with increasing *E*(*K*|*K*>0). Similar behavior could be observed in the school scenario; however, the plot was mirrored at a different conditional mean, and the white line with Δ*prob*=0 had a higher slope (Figure S15 in Multimedia Appendix 1).

In Figure 5B, the likelihood and priors with 2 different parameter sets that result in the same predicted probability are plotted. The likelihood had a maximum value of 1 for no further infections and decreased monotonically for higher numbers of newly infected persons. The priors had their maximum value also for no infection but with different behavior. The first prior had higher values for a smaller number of infected persons, whereas the second prior has higher values for >3 infected persons as the higher *E*(*K*|*K*>0), the more mass was shifted from the lower values toward the upper values.

The influence of the test sensitivity on the predicted probability was almost linear, with a slope of approximately 0.2 (Figure S16 in Multimedia Appendix 1).

### Usability Evaluation

#### Cognitive Walk-Through

We conducted the cognitive walk-through in the local health authorities of Berlin-Reinickendorf and Bremerhaven with 3 containment scouts each. The users tested the web application version as of May 2022.

Most of the testers (4/6, 67%) were able to complete tasks 1 to 3, apart from testers 2 and 6, who did not manage to apply the task to the tools. Even for the testers who were able to complete the task, the results of task 2 were confusing as the graphic was not intuitive and was described using misleading terms. Tasks 4 and 5 were completed only by testers 4 and 5. For the other testers, the input was not intuitive; therefore, they were not able to complete tasks 4 and 5.

**Figure 6:**
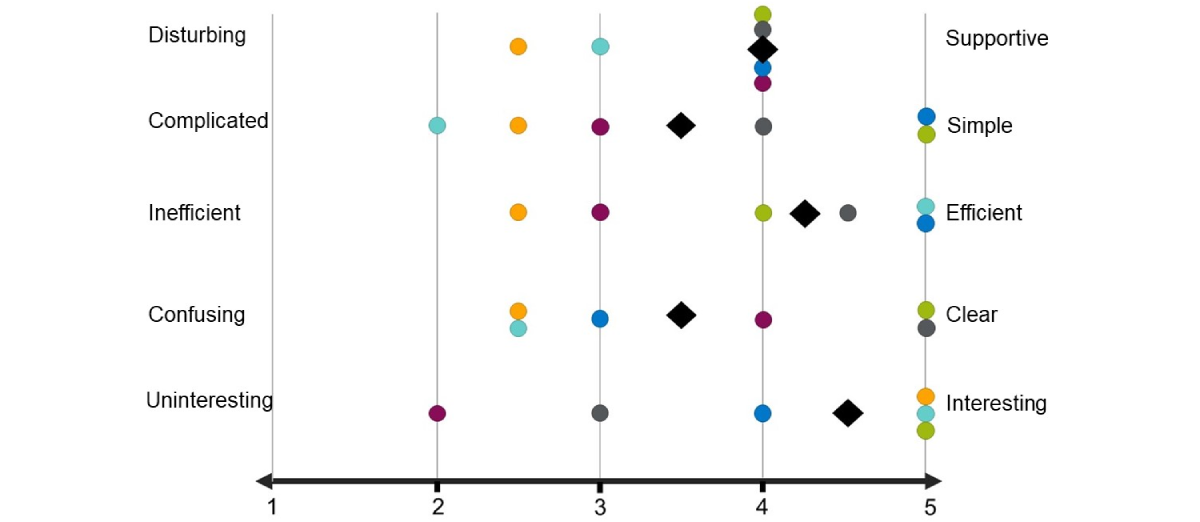
Ratings of the web application on a 5-point Likert scale, with 1 indicating full agreement with the negative attribute and 5 indicating full agreement with the positive attribute. The 6 testers are indicated in (1) purple, (2) orange, (3) turquoise, (4) blue, (5) green, and (6) gray. The median is indicated as a black square.

In general, the users rated the web application as supporting and efficient but rather complicated and confusing when using it for the first time (Figure 6). Assuming a Likert scale from 1=*disturbing* to 5=*supporting*, the median of 4 refers to most users (4/6, 67%) rating the EsteR toolkit as supporting. Accordingly, the users found the web application to be more *efficient* than *inefficient* (median 4.25) and more interesting than *uninteresting* (median 4.5). Rating the web application as 1=*simple* to 5=*complicated* revealed heterogeneous results, with a tendency to perceive it as simple (median 3.5). On the Likert scale of 1=*clear* to 5=*confusing*, the users tended to find the front end clear (median 3.5). At least 33% (2/6) of the users each agreed in describing the EsteR toolkit as simple, efficient, clear, and interesting. None of the users stated that the web application was fully disturbing, complicated, inefficient, confusing, or uninteresting.

All users (6/6, 100%) stated that the visualization of the results was generally helpful, but some (4/6, 67%) found it hard to interpret. In particular, the interpretation of the 80% and 95% time intervals was unclear to the testers. Some testers (3/6, 50%) expected more detailed information on the event in question, especially for the last 2 tasks. For the testers who tried, it was not possible to generate a PDF document of the results shown in the web application. Nearly all users (5/6, 83%) stated that more guidance and experience would be helpful to use the toolkit efficiently.

The testers indicated that they would adapt the tools for the risk assessment of diffuse events under (partly) unknown circumstances. Moreover, the containment scouts stated that the use cases might help manage groups of >3 people. The detailed results of the 6 cognitive walk-throughs are presented in Multimedia Appendix 2.

#### Focus Group Interviews

The interviews were conducted with focus groups in each of the local health authorities of Berlin-Reinickendorf and Bremerhaven. The 2 focus groups consisted of the 3 containment scouts from each local health authority who had previously tested the web application via the cognitive walk-through.

In general, the containment scouts were highly experienced in assessing transmission risks when applying the official guidelines of the federal government agency RKI. The scouts emphasized that they assessed individual cases and, therefore, valued the personal contact with the index case and contact persons via telephone. In this way, they normally gathered detailed information about the event in question. This workflow is time-consuming and could not be maintained during the entire course of the pandemic depending on local transmission clusters, current policies, and guidelines. Therefore, the containment scouts appreciated digital decision tools as they might help in handling the varying workload and human resources. They saw the potential of the web application to offer support, especially in justifying their decision on group quarantine as the toolkit provides a concrete specification of transmission risk. A total of 33% (2/6) of the testers saw the potential of using the web application to train new staff. Unexperienced containment scouts could insert known data and view time frames when transmission may have occurred.

The containment scouts raised concerns that using the web application during common workflows might be too time-consuming. They agreed on preferring short and intuitive features accompanied by less text and explanations. As a trade-off, the functionalities and results needed to be understood. Therefore, more training with the web application would be needed. In one focus group, the idea of a manual with basic explanations of functionalities and their interpretation was discussed.

All the containment scouts (6/6, 100%) were experienced in assessing transmission risk and periods. Most of them (4/6, 67%) expressed that the toolkit’s results were in agreement with their expectations based on experience. In total, 33% (2/6) of the containment scouts raised the concern that inexperienced colleagues could tend to rely on the web application’s results without questioning their plausibility.

A major concern was the dependence of local health authorities on policies and guidance from the RKI. The local health authorities needed to continuously adapt their daily work to the course of the pandemic. The containment scouts indicated that the web application might not fit their workflows at different stages. Nevertheless, they agreed that contact tracing might be more relevant in the future when the epidemiologic situation and policies change. Further potential was remarked for the first risk assessment for staff in childcare facilities and schools, as well as an adaptation to other infectious diseases.

## Discussion

### Sensitivity Analysis for Statistical Models

#### Principal Findings

##### Infection Period

In the written answers of our web application, the 80% HDRs were rounded to days. The default parameters led to a period of 8 days for the 80% HDR and a 1-IoU of <0.2, which corresponded in almost all cases to at most 2 days that are part of the union but not the intersection of two 80% HDRs. For example, the 80% HDR of point 1 in Figure 1 starts on the same day but ends 1 day after the 80% HDR from the default parameters. Thus, we rated the results as stable regarding the mean and median of the incubation time distribution in the area within the first contour line of 0.2. This area covered half of the studies (14/28, 50%) and especially included the 11% (3/28) of studies that were the most recent in terms of the time of data collection.

##### Infection Spread

In this use case, the results were more susceptible to parameter deviations than before as, in our underlying group scenario, the prediction was only influenced by the mass of the distribution of the first 4 days. This part of the distribution changed faster than the overall shape and the 80% HDRs when altering the mean or median (Figure 1B). In a different scenario, the deviations in the predicted infections could be even higher in both directions. Hence, it was not possible to determine a range of parameters for which the model is generally stable.

##### Illness Period

As for the infection period, we reported the 80% HDRs in our web application in days. In this case, the default values led to a period of 9 days, and a 1-IoU of <0.2 again implied for almost all cases a deviation of 2 days at the most. For example, the 80% HDR for point 1 in Figure 3 actually starts and ends on the same day as the one from the default values. As we expected the first generation to be of most interest, we rated the model to be stable regarding the mean and SD of the serial interval in the area within the contour line of 0.2 of the 1-IoU plot of the first contact generation. This area was only slightly smaller than the one from the contour line of *W*_1_=1 in Figure 3A and covered approximately half (17/35, 49%) of the studies included for this use case.

##### Infectious Period

In the web application, the default values resulted in a reported 80% HDR with a length of 8 days. For a 1-IoU of <0.2, the difference between two 80% HDRs was again, in most cases, ≤2 days. Hence, we again rated the model to be stable regarding the mean and SD in the area within the first contour line of 0.2. Unfortunately, as we were not able to identify other studies that modeled the infectious period, we could not rate the stability of our study selection.

##### Risk Assessment for Group Quarantine

The nonmonotone course of the prediction when increasing the *E*(*K*|*K*>0) with a fixed *P*(*K*>0), as shown in Figure 5A, is due to the complex relationships inside our model: the prediction is derived from the prior distribution for the COVID-19 transmission model for the specific group situations and a likelihood representing the current test situation in the group. In the simulations, the prior was determined by *P*(*K*>0) and *E*(*K*|*K*>0), whereas the likelihood remained unchanged as it depended on the number of negative tests per day and the sensitivity of the conducted test types, which were fixed.

To calculate the probability of no further infections, the prior probability for *K*=0 was divided by the sum of the product of the prior and likelihood. The mass shift in the prior distribution between points 1 and 2 in Figure 5 was caused by the higher *E*(*K*|*K*>0) and resulted in the same probability for the 2 parameter sets in this specific group and test situation. The school scenario was simulated in the same parameter range but with another group and test situation and resulted in a different mirrored behavior than that of the childcare scenario. Thus, it was not possible to determine a range for the model parameters for which the resulting probability is stable as the behavior highly depended on the specific group setting.

The variation in the day-dependent test sensitivity shows that it has only a low influence on the predictions, and the model is stable regarding this sensitivity.

#### Limitations

For each use case, we created a single scenario defined by the input data for which we conducted our simulations. However, many more combinations of different input data could be chosen for the use cases of the infection period for more than one person, the infection spread, and the risk assessment for group quarantine. For the sensitivity analysis, considering all possible combinations would increase the complexity of the simulation and make the interpretation of the results more challenging. Thus, we decided to analyze the infection period only for one person, and we created group situations we typically expect as user input for the infection spread and the risk assessment of group quarantine. Nevertheless, some sensitivity effects might be missed.

Furthermore, the literature review was from March 2022 and resulted in papers that mainly analyzed data from early 2020. Thus, work published since March 2022 is not included in the review or in our analysis. For the infectious period and the risk assessment for group quarantine, we even identified no further studies and, thus, had to define plausible model parameter ranges for the sensitivity analysis on our own.

#### Comparison With Prior Work

Sensitivity analysis is a widely used tool, and in the literature, it has been used for various COVID-19–related research questions (eg, it has been used to analyze epidemiological models [5,6,8] or understand the transmission of COVID-19 [7]). Weng and Yi [9] conducted a sensitivity analysis to estimate the COVID-19 incubation time based on a systematic literature review. The results of the sensitivity analysis showed that the incubation time varied when excluding or including certain studies. Our statistical model for the infection period and spread was also based on the incubation period. Our data obtained from a literature review [14] also showed a high heterogeneity.

### Usability Evaluation

#### Principal Findings

##### Cognitive Walk-Through

The cognitive walk-throughs revealed critical issues of the web application and users’ understanding of features and results. The concerns and misunderstandings gathered led to an update of features of the web application.

In this study, the cognitive walk-throughs showed that the tools were not easy to understand as not all testers were able to complete the example tasks that included basic information on containment. In total, the participants in our study provided similar feedback that varied according to their personal attitudes toward digital tools in general and their experience with containment. In particular, use cases 4 and 5 on group meetings were not intuitive. Accordingly, the input UI was restructured in such a way that the inputs are aligned with the tools. Furthermore, the testers’ feedback showed that the results statements and graphics could be misunderstood or not understood at all. The visualization of the results was generally stated to be helpful, and some testers (2/6, 33%) highlighted that it should be as easy to understand as possible, either self-explanatory or accompanied by a brief explanation. Thus, we added more detailed descriptions to the graphics and explanatory texts. On request, the results section of each tool in the web application provides an example of how to interpret the calculated probabilities.

The cognitive walk-throughs disclosed functionalities that were not working properly and minor bugs. This led to adaptations of the back end. The function to create a PDF document of the results shown in the web application was not working. Thus, this function was removed from the UI.

Most of the testers (5/6, 83%) expected to be able to use the tools properly after trying out some more inputs. However, more guidance would be appreciated. The wording used to guide users in inserting the inputs was adapted. The UI of use cases 4 and 5 was adapted with a clear workflow to enter the inputs and more detailed descriptions of the inputs and results. All users were highly experienced in COVID-19 risk assessment and used their expertise to interpret the results of the tools. Usually, the containment scouts process detailed information on the event in question for a more precise risk assessment. We discussed the number of possible inputs to the models and decided to provide general tools that are applicable to various events. In general, the containment scouts stated that the tools might be helpful in justifying their decision on whether to order quarantine for contact persons.

##### Focus Group Interviews

The feedback from the focus group interviews showed the containment scouts’ perception of the values and concerns regarding the web application and revealed suggestions to improve usability. The suggestions were implemented when applicable within the tools and relevant for the broader purpose of the toolkit.

In the focus group interviews, the containment scouts were open and interested in integrating the digital decision support tool into their workflows when suitable. The scouts highlighted the importance of efficient contact tracing to contain COVID-19 outbreaks. Most of them (5/6, 83%) stated that they would use the EsteR toolkit or recommend it to colleagues in case the policies changed and set contact tracing back in the focus of pandemic control. Tools of the web application should have a high comprehensibility to allow for use in daily work by local health authorities. This indicates that the UI should be as simple and clear as possible. In this way, the web application can also serve as an efficient tool instead of being unnecessarily time-consuming, which some containment scouts were concerned about. The apprehension that the tools might be too time-consuming was already expected beforehand as we consulted an executive of a local health authority regularly while developing the web application.

Another important concern is the rapidly changing policies of the RKI. The statistical models behind the web application are based on the COVID-19 transmission properties obtained from our latest literature research [14]. The tool for calculating the infectiousness period of persons without symptoms was based on the RKI policies until May 2, 2022, which are displayed in the results of the tool. At the time of the interviews, contact tracing was not a priority for pandemic containment. The local health authorities were not in charge of ordering quarantine for suspected infected persons. Therefore, the containment scouts highlighted that the web application should be as flexible as possible to adhere to changing RKI regulations. The tools are generally based on the evidence found in international peer-reviewed literature. In this way, single tools can be adapted and applied to specific cases.

The target group was rather heterogeneous in terms of experience with knowledge of COVID-19 transmission risks and with digital tools. In the interviews, the containment scouts discussed the idea of using the web application to familiarize new employees with the work tasks and support the decision-making process. To address this suggestion, we simplified the tools in the web application and added more detailed descriptions and helpful explanations on request. In this way, the web application can be used intuitively for the first time without reading a manual or receiving further training.

#### Limitations

We expected more differential feedback for the use of the web application as each local health authority in Germany works slightly differently. Only 6 containment scouts from 2 local health authorities participated in the cognitive walk-through. Unfortunately, it was not possible to reach out to another local health authority for the usability evaluation.

Furthermore, user feedback was limited to their first impressions and whether they could solve the tasks on the first or second try. Related to the feedback that they would prefer to try out some of the web application functionalities intuitively and get an impression of how to use it, we will provide the web application to the users before conducting the cognitive walk-throughs next time. We would expect more specific feedback and further thoughts on implementation after users have already tried it out. However, users should not already be familiar with the web application to gather their perceptions for intuitive use.

Furthermore, the usability evaluation was limited to only 6 participants from 2 local health authorities.

Our results might be biased as the containment scouts we approached were highly experienced with contact tracing. Their perceptions of efficient decision support coincided with feedback on guidance and explanations. Nevertheless, the participants had different backgrounds using digital tools. Therefore, we assessed different levels of expertise in working with the UI. However, a major limitation is that the containment scouts were not mainly working on contact tracing at the time of the interview owing to the currently valid regulations. By that time, the RKI policy did not focus on contact tracing, and persons suspected of being infected were quarantined. Therefore, the containment scouts had to think back to the time when they had to justify their decision on whether to order quarantine. Nevertheless, they expected the regulations to be adapted to the course of the pandemic, so contact tracing might be important again during times of higher infection numbers or higher hospitalization rates.

#### Comparison With Prior Work

Although the cognitive walk-through method has been applied for UI evaluation of digital applications in general [79], it has recently been applied to digital applications targeting health outcomes. For example, user-centered cognitive walk-throughs have been applied to evaluate a diabetes self-management tool. This method has been shown to be sensitive in identifying critical usability problems and generating recommendations to improve the effectiveness, efficiency, and user acceptance of the tool [10]. Moreover, a decision support tool in the clinical context of self-medication has recently been evaluated through cognitive walk-throughs. Dargan [11] found that their tool was generally understandable and helpful for users. The participants provided recommendations to improve content and layout that the developers could adapt to improve the usability and satisfaction of the decision support tool.

Focus group interviews have been applied for usability evaluation (eg, of clinical decision support tools [12,13]). Kastner et al [12] assessed physicians’ attitudes toward an osteoporosis disease management tool and consequently designed a functional prototype of the tool. The interviews revealed the importance of the use of the tool being as time-saving and handy as possible, as well as specific recommendations for features to be implemented. Ahearn and Kerr [13] conducted focus group interviews to explore the intended users’ attitudes and expectations for integrating such tools into their workflows. The participants stated that the output should be as narrow as possible to not overwhelm the user with less important information.

Accordingly, in our study, we identified functionalities that were not working properly and features that led to misuse and misunderstanding, obtained recommendations for layout improvements, and obtained feedback that the web application would help familiarize the user with the context and support the decision-making process. The containment scouts highlighted the importance of efficient contact tracing to contain COVID-19 outbreaks. We assessed similar concerns as in the focus group interviews conducted by Kastner et al [12] and Ahearn and Kerr [13], namely, that the features need to be easy and fast to use to implement the tool in the workflow.

### Conclusions

In this study, we validated every statistical model of the EsteR toolkit through a sensitivity analysis of the model parameters derived from a literature review [14]. The results of this simulation study revealed how stable the statistical models used are and, thus, how carefully their parameters must be chosen. For the single infected person use cases (infection period, illness period, and infectious period), we were able to identify ranges for all model parameters for which the results were stable. However, the infection spread for groups was very sensitive to parameter changes, and this use case, as well as the risk assessment of group quarantine, highly depended on the specific group setting. These 2 use cases would benefit the most from an update of the model parameters to the exact situation at hand. Furthermore, the data sets provided by the literature review showed a high heterogeneity.

In the second step, we conducted cognitive walk-throughs and focus group interviews with containment scouts to evaluate the usability of the web application. On the basis of the results, the UI of the web application was adapted to make it more intuitive and easier to learn and reduce the time of use.

For now, the EsteR toolkit will not be improved further as the COVID-19 pandemic does not play a major role for local health authorities anymore. However, in the case of a new pandemic arising, the toolkit can be easily adapted and extended as soon as studies providing pathogen properties such as incubation time are published. This evaluation study quantifies the general usefulness of the EsteR toolkit and demonstrates the extensive evaluation of a decision support tool in the public health sector, providing all data and code for reproducing the sensitivity analysis.

## Appendix

Multimedia Appendix 1 Simulation report.

Multimedia Appendix 2 Results of the cognitive walk-throughs.

## Abbreviations

HDR: high-density region
IoU: intersection-over-union
RKI: Robert Koch Institute
UI: user interface

## Data Availability

The analyzed data extracted from the included studies on incubation time and serial interval and the code used for the sensitivity analysis are available in the GitHub repository [75].
