# Supplementary material for: Evaluation of the EsteR Toolkit for COVID-19 Decision Support: Sensitivity Analysis and Usability Study
Source: JMIR Form Res. 2023 Jun 27;7:e44549. doi: 10.2196/44549 (PMC10337391; doi:10.2196/44549)
Supplement: Multimedia Appendix 1 [file formative_v7i1e44549_app1.pdf]

Multimedia Appendix 1: Simulation Report

This document is an appendix to the paper Alpers et al. 2023: *Evaluation of the EsteR Toolkit for COVID-19 Decision Support: Sensitivity Analysis and Usability Study*, JMIR Formative Research.

1 Selection of Default Parameters for our Web Application

1.1 Incubation time for infection period and infection spread

In the literature review described in [14], 123 studies reported aspects of the incubation time of a COVID-19 infection. Some of them mentioned fitting a distribution to their data and if so, it was most often a lognormal distribution. Hence, we decided to use a lognormal distribution for the incubation time used in the use cases infection period and infection spread. Because only four studies directly reported the lognormal distribution parameters, we decided to derive them on our own from all studies which reported a mean and median incubation time, as there is a direct link between the mean and median of a lognormal distribution and its distribution parameters μ and σ. Even though 32 studies reported a mean and median, only 28 of them could be used to calculate a lognormal distribution, because there the mean is always larger than the median.

As we are using the lognormal distribution parameters directly in our web application, we made the decision for a final parameter set based on μ and σ and not on the mean and median incubation time. In Figure S1 we can see the derived parameter sets from all 28 included studies [15, 19-45]. Because only three studies contain data from later than the first half of 2020 and they all do not show conspicuous behavior compared to the other studies, the information of study data collection time did not contribute to our decision. Instead, we calculated the mean squared distance for every parameter set to every other set and chose the parameters with the minimal distance. This set is marked in red and corresponds to the study from Xin et al 2020 [15].

1.2 Serial interval for illness period

In the literature review described in [14], 95 studies reported aspects of the serial interval of a COVID-19 infection. Some of them mentioned fitting a distribution to their data and if so, it was most often a gamma distribution. Hence, we decided to use a gamma distribution for the serial interval used in the illness period use case. Because only five studies directly reported the gamma distribution parameters, we decided to derive them on our own from all studies which reported a mean and standard deviation of the serial interval, as there is a direct link between the mean and standard deviation of a gamma distribution and its distribution parameters α and β. Even though 47 studies reported a mean and standard deviation, only 35 of them could be used to calculate a gamma distribution. If the mean is smaller than the standard deviation, the distribution would approach infinity on the

symptom begin date of the index case, which does not make sense for our model. By choosing a gamma distribution with mean larger than the standard deviation, we implicitly make the assumption that the serial interval is non-negative.

As we are using the gamma distribution parameters directly in our web-application, we made the decision for a final parameter set based on α and β and not on the mean and standard deviation of the serial interval. In Figure S2 we can see the derived parameter sets from all 35 included studies [16, 19, 30, 34, 36, 44-73]. Again, most of the underlying data was gathered in the beginning of the pandemic and thus the time of data collection did not contribute to our decision. On the lower left part of the plot, a cluster of parameter sets is observable. No outliers can be found below the cluster due to our implicit modeling assumption that the serial interval is strictly positive. Hence, we decided to also invoke an upper bound and exclude the ten studies with the highest α and β values from the decision for a final parameter set. Inside the cluster we again calculated the mean squared distance from each parameter set to all other sets of the cluster and chose the parameters with the minimal distance. This set is marked in red and corresponds to the study from Son et al 2020 [16].

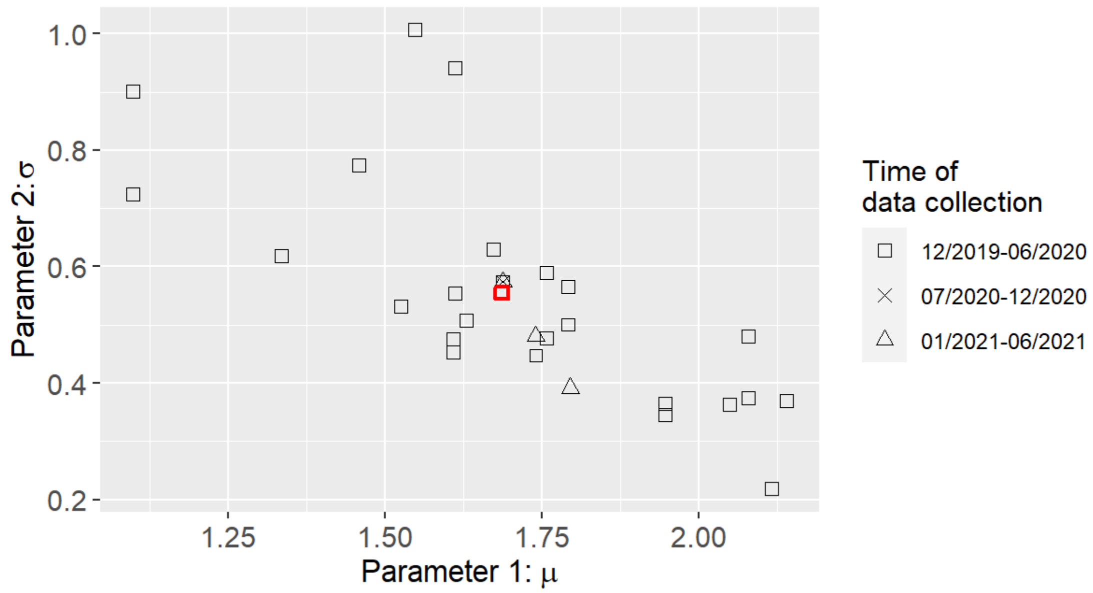

Figure S1: All lognormal distribution parameter sets derived from the included studies for the incubation time. The reference parameters are marked in red.

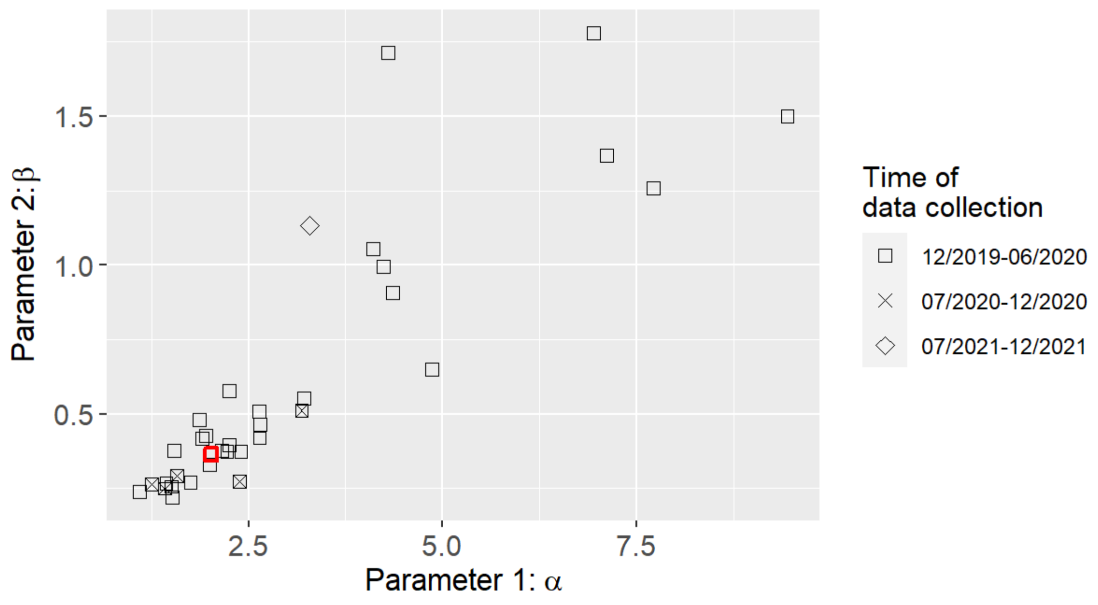

Figure S2: All gamma distribution parameter sets derived from the included studies for the serial interval. The reference parameters are marked in red.

1.3 Infectious period

Even though four studies were identified in [reference (8) in the main paper] that reported aspects of the infectious period, only the study from He et al 2020 [17] where they fit a gamma distribution with shape parameter $\alpha = 20.516508$ and rate parameter $\beta = 1.592124$ was suitable for our modeling approach. Hence we directly use those parameters in our EsteR toolkit.

1.4 Setting-specific transmission and PCR-test sensitivity for group quarantine

In our Bayesian modeling approach for the risk assessment of group quarantine use case, the beta binomial distribution parameters α and β of the prior are derived from the probability $P(K > 0)$ that K persons are infected with COVID-19 and the conditional mean number of infected persons in the case of a transmission $E(K|K > 0)$. Inside the EsteR toolkit, the user can choose between a childcare group setting and a school class setting. The respective default parameters are taken from Schoeps et al 2021 [18] as follows: In the childcare setting, we directly utilize the reported values for children (0-6 years) in day-care. Contrary to our statistical model, in [18] they differentiate between three different school types, so we decided to take the respective means from all three types for our school setting.

For the day-dependent PCR-test sensitivity, which is used in the likelihood of our model, we directly use the values reported in Kucirka et al 2020 [74].

2 Details Regarding the Simulation Strategy

2.1 Infection Period

The infection period is based on the incubation time modeled with a lognormal distribution as described above in chapter A.1. Specifically, the default values for the lognormal distribution parameters in our web application are $\mu = 1.69$ and $\sigma = 0.55$ derived from a reported mean of 6.3 and median of 5.4 of the incubation time in [15]. To test the influence of the chosen mean and median incubation time on the model outcomes in our simulations in a plausible range, we set the respective minimum reported mean and median from the 28 included studies in A.1 as the lower and the maximum reported mean and median as the upper bounds. Inside both ranges we defined a sequence of 100 equidistant values each and used all suitable parameter combinations from those sequences to calculate a lognormal distribution and determine the resulting distribution and the 80% HDRs for the infection period of one person starting to show symptoms on March 22nd, 2022. Not all possible combinations were suitable, because for a lognormal distribution the mean is always larger than the median.

In the EsteR toolkit, it is also possible to calculate infection periods for more than one person. We decided to test a scenario with only one infected person because it is probably of more interest to potential users and the 80% HDRs are easier to interpret.

2.2 Infection Spread

In the infection spread after a group event use case is built upon the same incubation time distribution as the infection period. Hence, we tested the same default parameters and parameter ranges than before. We worked with a scenario of 20 persons who met on March 22nd, 2022. Up to March 26th, 2022 three of them reported beginning to show symptoms of a COVID-19 infection. With this input, the model predicts the total number of symptomatic infections one can expect from this event.

As in the web application we do not restrict this use case to certain settings, the group size, the number of observed illnesses and the number of days after the event the last illness was observed could have been chosen arbitrarily for the simulation. However, we tried to define a scenario we expect to be typical to assess in a local health authority. We assume that mostly small to medium groups (e.g. a birthday party) will be of interest where the extent of infection needs to be estimated after the first few people develop signs of a COVID-19 infection.

2.3 Illness Period

The illness period is directly calculated from the serial interval which is modeled with a gamma distribution as described in chapter A.1. Specifically, the default values for the gamma distribution parameters in our web application are $\alpha = 2.02$ and $\beta = 0.36$ derived from a reported mean of 5.54 and standard deviation of 3.9 of the serial interval in [16]. To test the influence of the chosen underlying mean and standard deviation on the model in a plausible range, we set the respective minimum reported mean and standard deviation from the 35 initially included studies in A.1 as the lower and the maximum reported mean

and standard deviation as the upper bounds. Inside both ranges we defined a sequence of 100 equidistant values each and used all suitable parameter combinations from those sequences to calculate a gamma distribution and determine the resulting distribution and the 80% HDRs for the illness period of the first, second and third contact generation of one person starting to show symptoms on March 22nd, 2022. Not all possible combinations were suitable, because we assume for our gamma distributions that $\alpha > 1$ and hence the mean is always larger than the standard deviation.

2.4 Infectious Period

As there is only one study providing parameters for a gamma distribution of the infectious period, we needed to come up with plausible ranges to test the parameters in on our own. Because in the illness period the gamma distribution is tested regarding its mean and standard deviation, we used the shape parameter $\alpha = 20.516508$ and rate parameter $\beta = 1.592124$ reported in [17] to first calculate the corresponding mean of $E(X) = 12.89$ and standard deviation of $SD(X) = 2.84$. To test the influence of the mean and standard deviation on the model outcome, we decided to use a range of 8-18 for the mean and 1-5 for the standard deviation, because we hope that they cover most plausible parameter sets as they are similarly large as the ones from the illness period. We again defined an equidistant grid of 100 values each and used all possible combinations of model parameters to calculate the distribution and the 80% HDR for the infectious period of a person with symptom begin on March 22nd, 2022. Note that the script from [17] also provides a shift parameter we make use of in our web application, but as it only shifts the whole distribution and we are more interested in the changes to the shape of the distribution, we decided to not alter that parameter in our simulation.

2.5 Risk Assessment for Group Quarantine

The default values in the EsteR toolkit derived from [18] are a COVID-19 transmission probability of 0.3 and a conditional mean of 3.3 for the childcare group and a transmission probability of 0.12 and a conditional mean of 1.77 for the school class setting. The simulation of the transmission probability was conducted over the reported 95% confidence interval from for the childcare setting and over the union of the three 95% confidence intervals of the different schools from [18] for the school class setting. The conditional mean number of infected was tested in both settings in the range 1-8. We tried to define two scenarios with typical occurrences for Germany: On the one hand, a childcare group with 14 people plus one known infected person (so 15 in total), where seven people are tested negative with a PCR-test five days after the last meeting with the infected person; on the other hand, a school class with 27 people plus two known infected persons (so 29 in total), where ten of the 27 are tested with a PCR-test two days after the last meeting with the infected persons and six other people are tested once with a PCR-test two days and once with an Antigen-test six days after the last meeting. In both scenarios 100 equidistant values for both parameters are defined inside their respective ranges and all parameter combinations are used to calculate the probability of no further infections inside the respective groups.

We also conducted a sensitivity analysis for the diagnostic test sensitivity. We simulated a consistent shift in the range of –0.2 to 0.2 of the day-specific PCR-test sensitivity from [74]

over all days and calculated the probability of no further infections in the childcare scenario described above.

3 Results of the Sensitivity Analysis

For each simulation we drew a contour plot with the respective first parameter on the x-axis, the second parameter on the y-axis and the metric values displayed as different colors, with white around 0, orange in the positive part and blue in the negative part. Inside the plot we marked the default values currently used in the app which served as reference values in the metrics in red. In the three use cases infection period, infection spread after a group event and symptom onset period of contacts we also highlighted all parameter combinations extracted from literature with different shapes according to the time the underlying data was gathered.

3.1 Infection Period

The results of the 1-IoU of the 80% HDR for the infection period from Figure S3 are already discussed in detail in the main paper. Regarding the Wasserstein metric, comparing Figure S3 and S4, the areas of (relatively) high and low values match across most parts of the plot. Only towards the lower right the values of the Wasserstein metric increase more than for the 1-IoU. The absolute values of the metrics differ a lot, as the 1-IoU is naturally limited upwards by 1, whereas the Wasserstein metric can take arbitrary non-negative values, depending on how far away the masses of two distributions are.

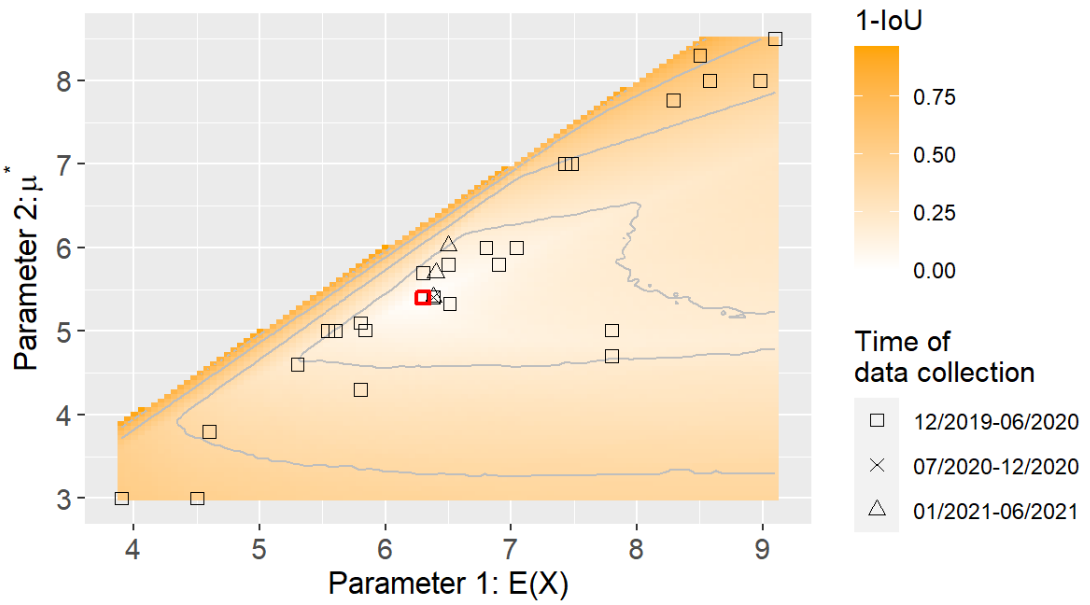

Figure S3: 1-IoU of the 80% HDR for the infection period.The reference parameters are marked in red.

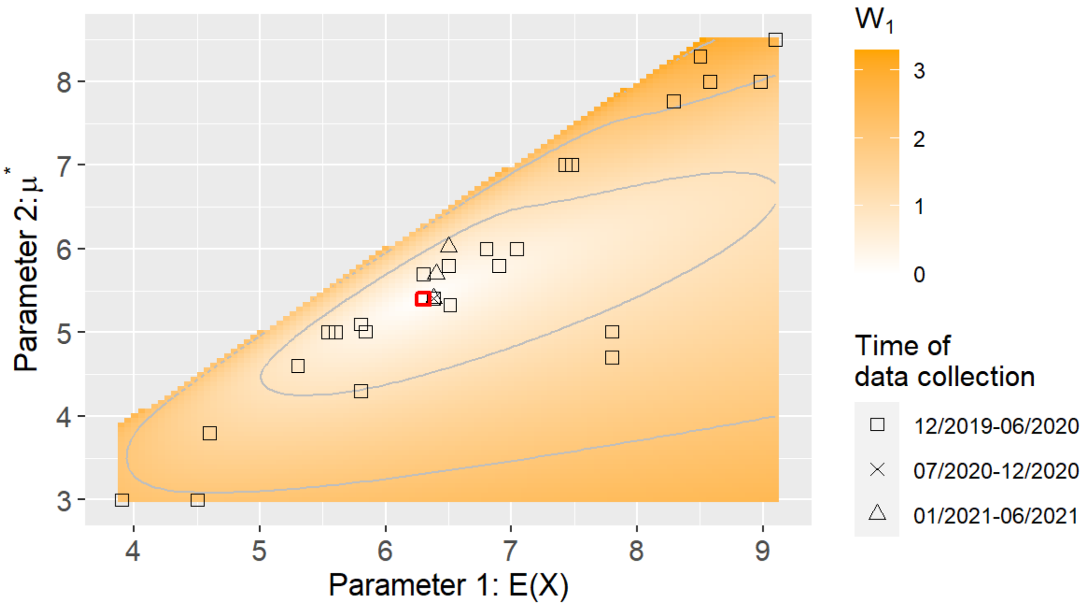

Figure S4: Wasserstein metric for the infection time distribution. The reference parameters are marked in red.

3.2 Infection Spread in Groups

The results for this use case in the specific scenario from Figure S5 are already discussed in detail in the main paper.

In general, the prediction in this use case mainly depends on the cumulative distribution function of the incubation time on the day the last symptom onset was observed (relative to the group event). We interpret that value as the fraction of observed infections compared to all infections that occurred at the event. The number of observed infection then leads to the prediction of the total number of infections, which is only limited upwards naturally by the total group size. If we had chosen a group size of e.g. 10000 instead of 20, the reference parameters would still predict a total of 11 infections and the minimal prediction in the simulations would still be 5, but the maximum predicted number of total infections in our simulations would be 10000. This new scenario may have given a better picture of the robustness of the incubation time distribution for our model. We decided against it because then our underlying assumptions that (a) all persons had only contact at the group event and (b) all observed infections occurred at that event are less likely to be fulfilled. Although it is not a hard condition, we expect our web-application to be mainly used for smaller groups.

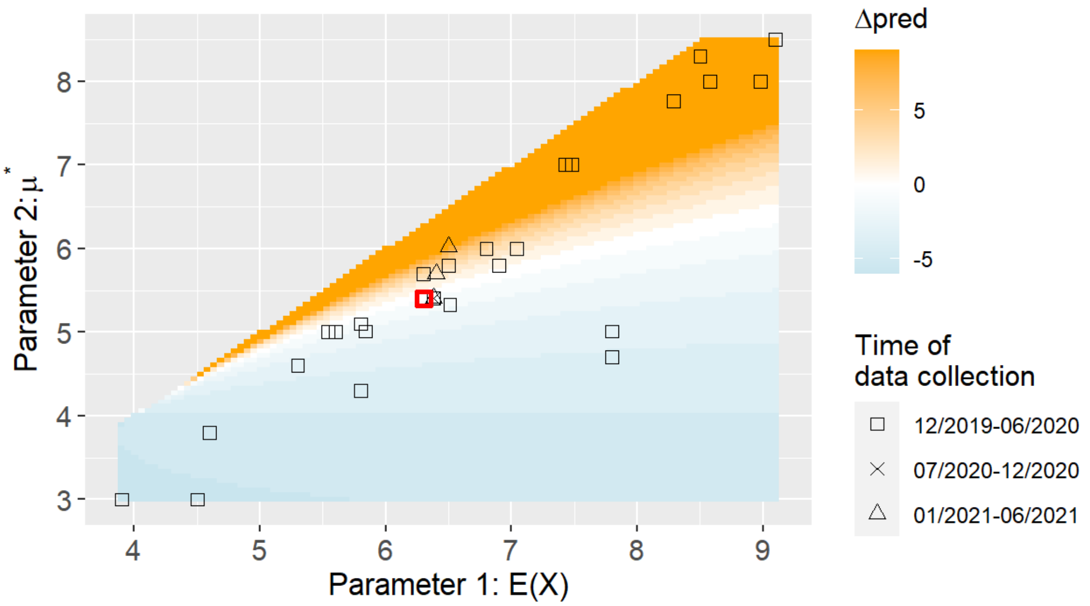

Figure S5: Difference of predicted total infections after a group event. The reference parameters are marked in red.

3.3 Illness Period of Contacts

The results of the Wasserstein metric for the illness period of the first generation of contacts from Figure S9 are already discussed and compared to the results from the second and third generation in Figure S10 and S11 in the main paper. The 1-IoU of the 80% HDR for the illness periods also shares areas of (relatively) high and low values across all three contact generations, as can be seen in Figure S6-S8. Compared to the Wasserstein metric, instead of an oval shape stretched along a vertical axis, the area of low values also follows a second axis diagonally from the bottom left to the top right, creating a pyramidal shape for a ll three generations. With increasing generations, the pyramid becomes more narrow and the metric values slightly increase.

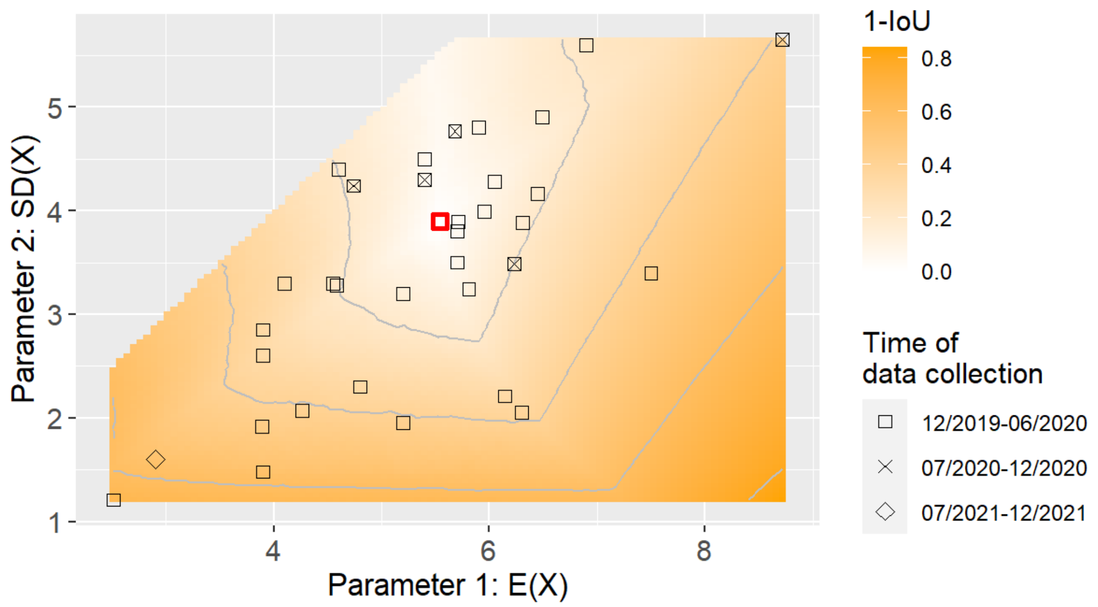

Figure S6: 1-IoU of the 80% HDR for the symptom begin of the first contact generation. The reference parameters are marked in red.

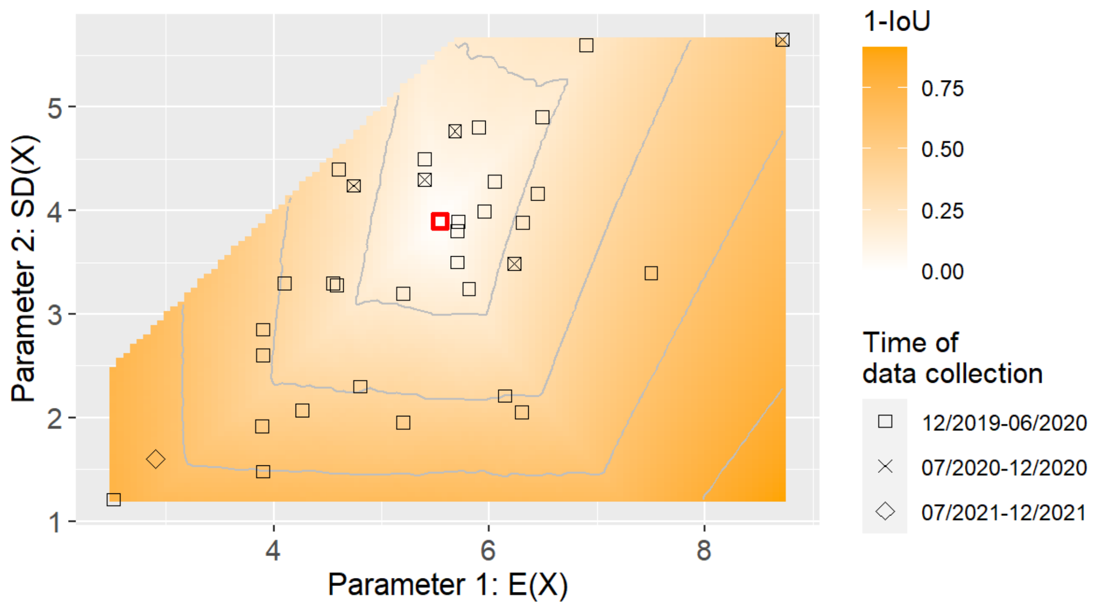

Figure S7: 1-IoU of the 80% HDR for the symptom begin of the second contact generation. The reference parameters are marked in red.

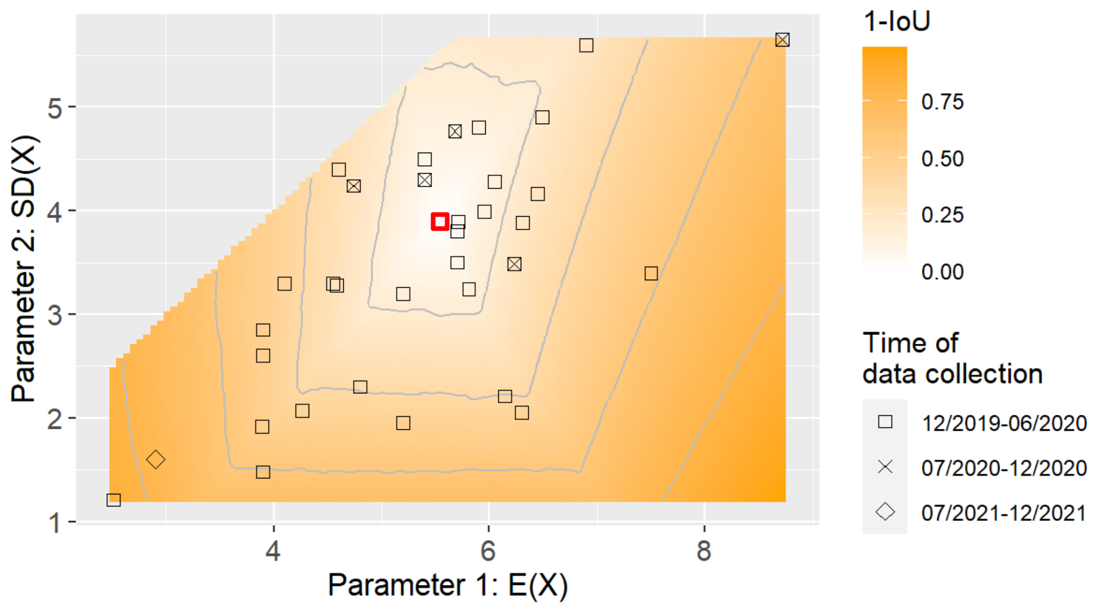

Figure S8: 1-IoU of the 80% HDR for the symptom begin of the third contact generation. The reference parameters are marked in red.

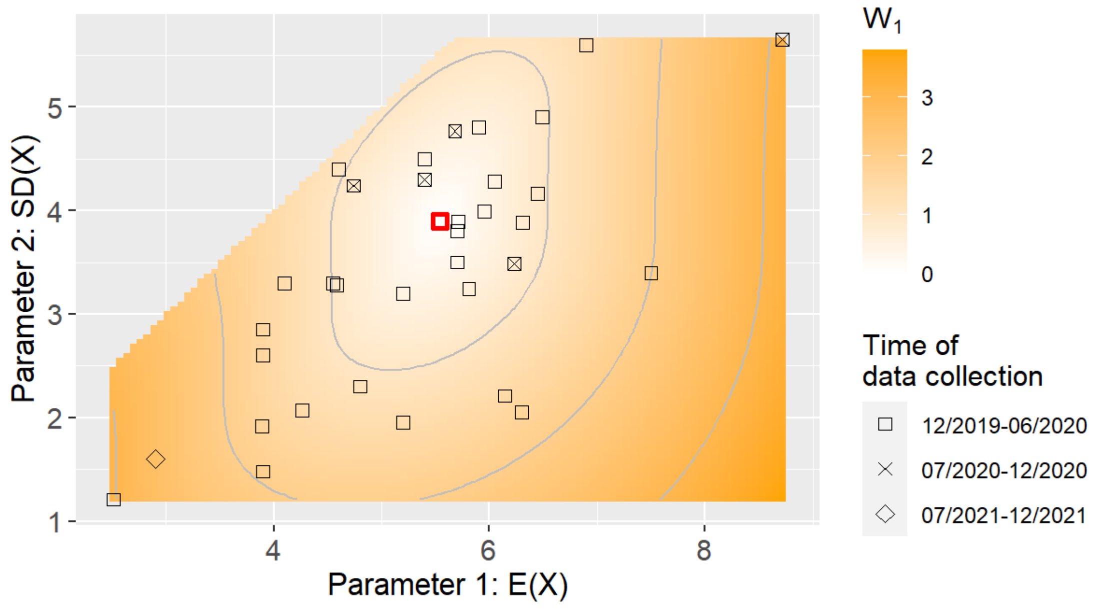

Figure S9: Wasserstein metric for the distribution of the symptom begin of the first contact generation. The reference parameters are marked in red.

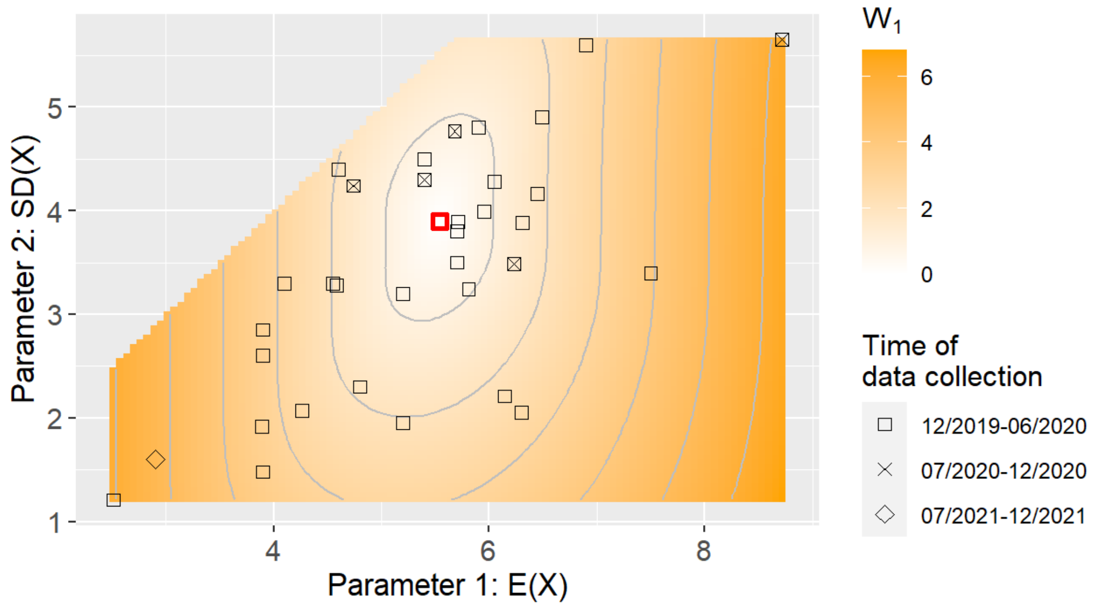

Figure S10: Wasserstein metric for the distribution of the symptom begin of the second contact generation. The reference parameters are marked in red.

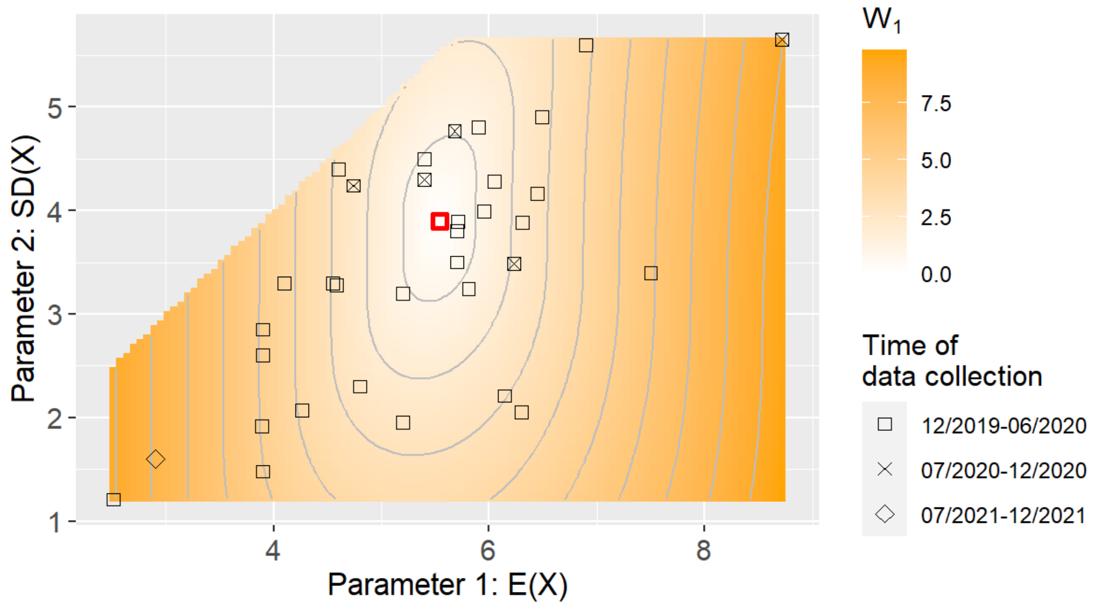

Figure S11: Wasserstein metric for the distribution of the symptom begin of the third contact generation. The reference parameters are marked in red.

3.4 Infectious Period

As already explained in the main paper, the results for the infectious period are very similar to the results from the illness period. The slightly tilted axes of the pyramidal shape for the 1-IoU and the different maximum values for both metrics seen in Figure S12 and S13 are probably due to the higher underlying means of the distributions in the simulation.

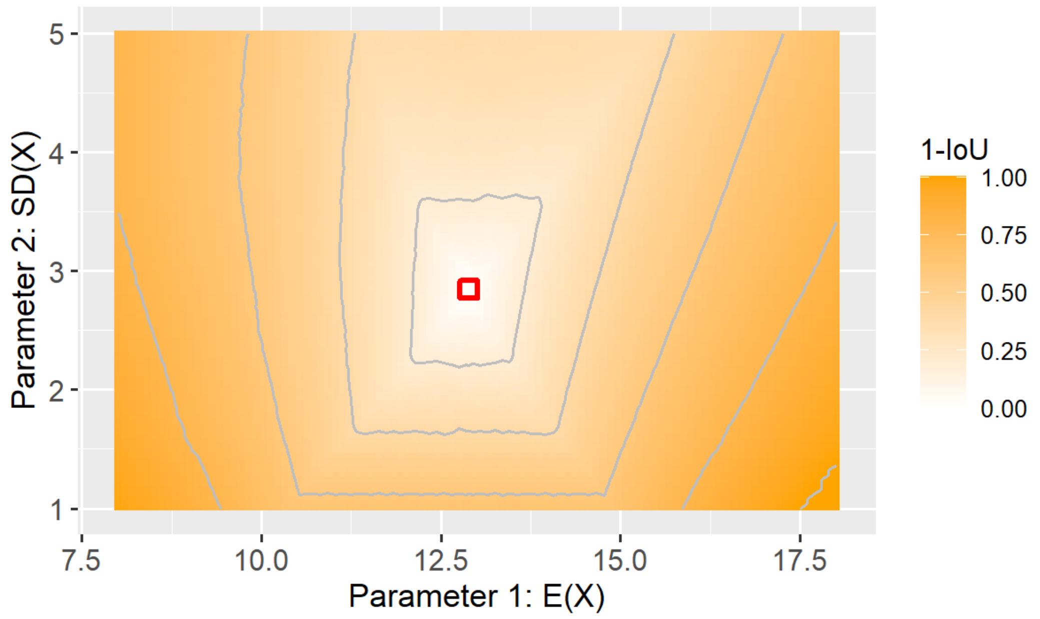

Figure S12: 1-IoU of the 80% HDR for the infectious period. The reference parameters are marked in red.

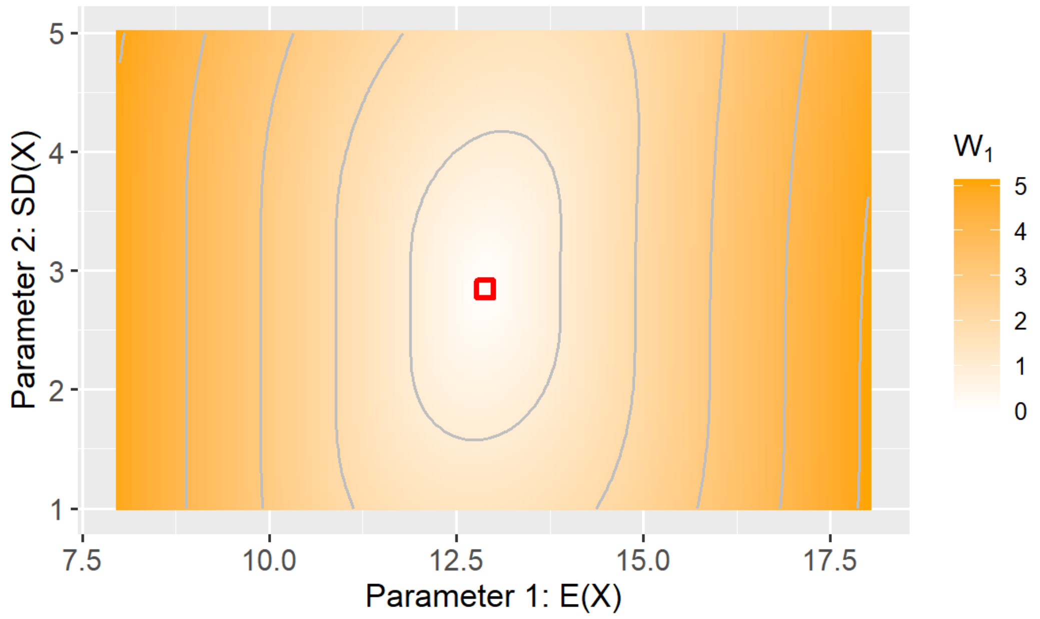

Figure S13: Wasserstein metric for the infectious period distribution. The reference parameters are marked in red.

3.5 Risk Assessment for Group Quarantine

The results of the probability difference in the childcare scenario in Figure S14 are already discussed in the main paper. For the school scenario in Figure S15, the course of the probabilities seems to be stretched vertically such that the mirror lines are now at $E(K|K > 0) \approx 1.1$ and 7.5 instead of 1.5 and 4.7, and the slope of the line of unchanged predictions almost doubled.

Comparing the results from childcare and school, it becomes clear that the course of the predictions is highly influenced by the input data from each scenario. If we had chosen the same group size, number of index cases, and number and dates of negative tests, both figures would simply show parts of the same plot where only the colors are shifted, as different reference parameters are used. Contrary to that, in our simulations Figure S14 and 15 show clearly different patterns of the predicted probabilities.

Figure S16 shows that the PCR-test sensitivity has only a minor influence on the predicted probability, as a change of 0.2 in both directions only leads to probability deviations of below 0.04.

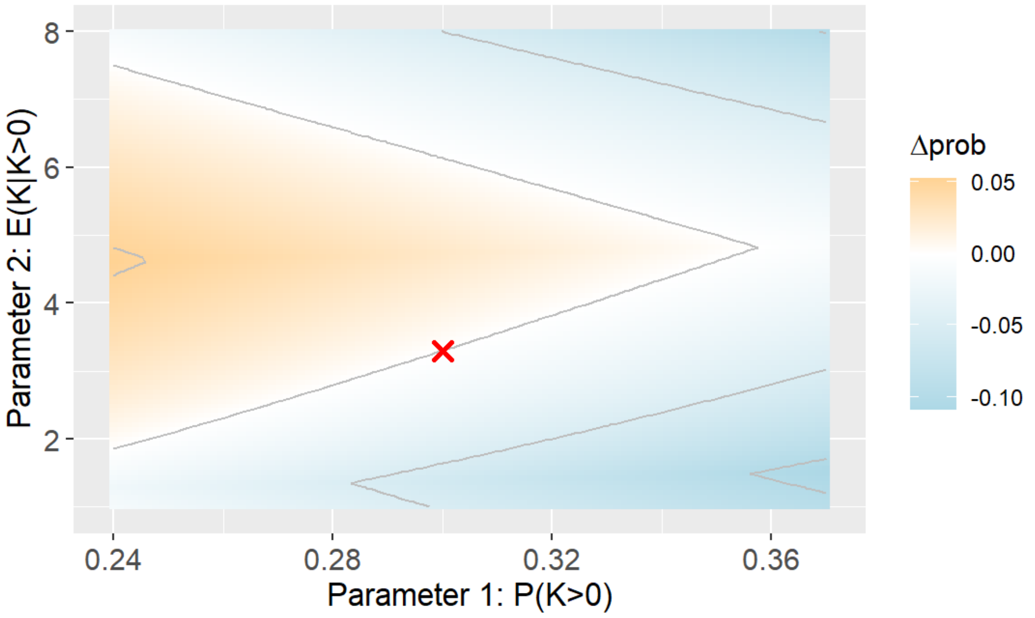

Figure S14: Difference in probability in the childcare scenario. The reference parameters are marked in red.

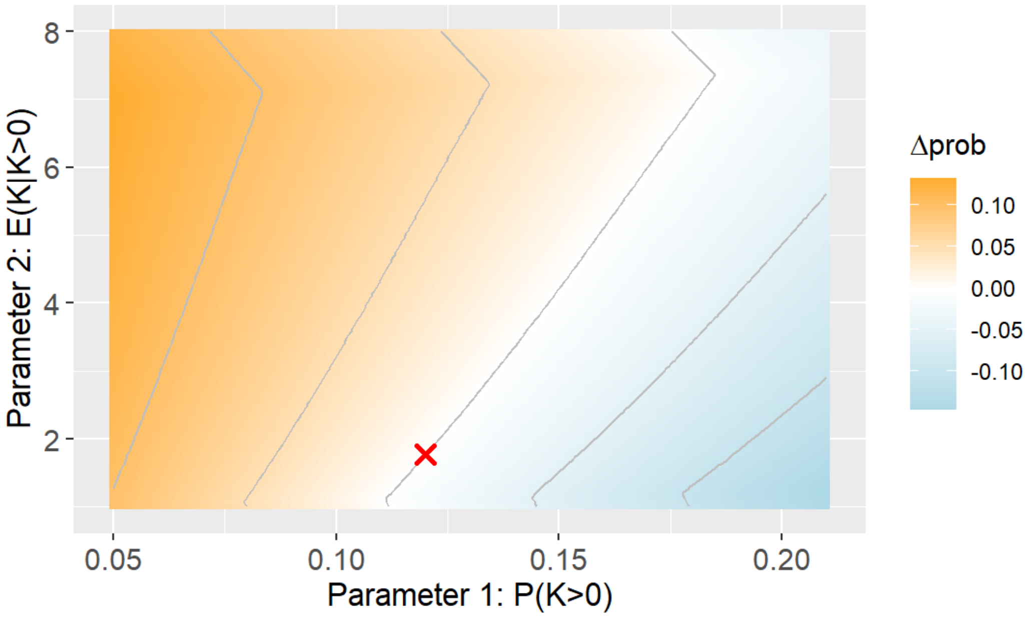

Figure S15: Difference in probability in the school scenario. The reference parameters are marked in red.

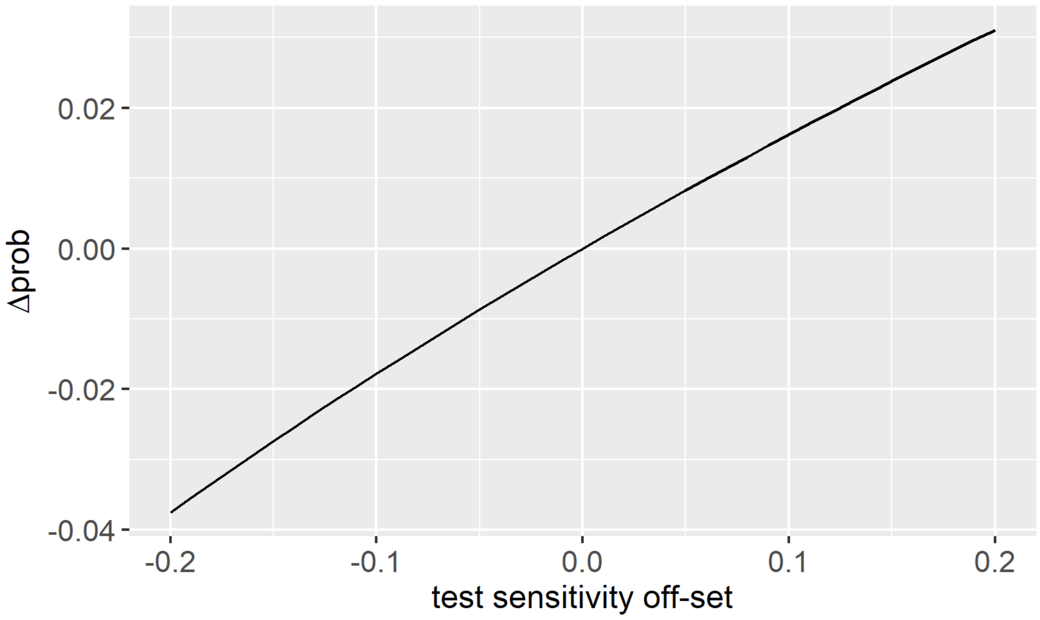

Figure S16: Difference of probability resulting from a shift of the PCR-test sensitivity.
