# Supplementary material for: Evaluation of the EsteR Toolkit for COVID-19 Decision Support: Sensitivity Analysis and Usability Study
Source: JMIR Form Res. 2023 Jun 27;7:e44549. doi: 10.2196/44549 (PMC10337391; doi:10.2196/44549)
Supplement: Multimedia Appendix 2 [file formative_v7i1e44549_app2.pdf]

Multimedia Appendix 2: Results of the Cognitive Walkthrough

This document is an appendix to the paper Alpers et al. 2023: Evaluation of the EsteR Toolkit for COVID-19 Decision Support: Sensitivity Analysis and Usability Study, JMIR Formative Research.

Table S1. Results of the cognitive walkthroughs of the five example tasks conducted by the six testers.

	Testers					
	1	2	3	4	5	6
Task						
1	• Input is clear<br>• Graph and interval colors are confusing<br>• Conforms to RKI policies	• More event details expected<br>• Assesses risk him/herself, not fitting to tool	• Input and result are clear<br>• Result is in line with expertise	• Fast orientation<br>• Input is clear<br>• Directly reads result, is hard interpret	• Fast orientation on page<br>• Input and result are clear	• Does not enter input<br>• Reads default result
2	• 80/95% intervals of time periods are confusing<br>• In accordance with RKI guidelines	• Does not enter input<br>• Reads default result<br>• Assesses risk him/herself	• Misleading term “generation”<br>• Result does not fit with expertise<br>• Cannot interpret result<br>• More training needed	• Input is clear<br>• Directly reads result, in line with expertise<br>• Figure gives fast info<br>• Misleading term “generation”	• Input is clear<br>• Directly reads result, in line with expertise<br>• Misleading term “generation”	• Does not enter input<br>• Reads default result
3	• More event details expected<br>• In line with expertise according to RKI guidelines	• Does not enter input<br>• Reads de-fault result	• Time periods are clear	• Input is clear<br>• Directly reads result<br>• 80/95% intervals were not understood	• Input is clear<br>• Directly reads result	• Does not enter input<br>• Reads default result<br>• More event details expected

		• Assesses risk according to expertise				
4	• Input is unclear<br>• Misleading term "children"<br>• More event details expected<br>• According to expertise	• Input is unclear<br>• UI too complicated<br>• Not able to fulfil task	• Interpretation according to expertise<br>• Uncertain in interpreting figure<br>• Concrete probability is helpful for decision	• Input is clear<br>• Result in line with expertise	• Input is clear<br>• Directly reads result<br>• Clearly interprets figure<br>• More event details expected	• Does not enter input<br>• Assesses risk him/herself
5	• Input is unclear<br>• Unclear when the persons were tested<br>• Unclear visualized timeline	• Input is not clear, especially for two testing days<br>• Not able to add contact event manually	• Input is unclear<br>• More training would be necessary	• Adding event is unintuitive, misleading<br>• Visualized timeline is helpful<br>• Interprets probability	• Input is clear<br>• Directly reads result	• Does not enter input<br>• Assesses risk him/herself
